# Supercell program: a combinatorial structure-generation approach for the local-level modeling of atomic substitutions and partial occupancies in crystals

**DOI:** 10.1186/s13321-016-0129-3

**Published:** 2016-03-31

**Authors:** Kirill Okhotnikov, Thibault Charpentier, Sylvian Cadars

**Affiliations:** NIMBE, CEA, CNRS, Université Paris-Saclay, CEA Saclay, 91191 Gif-sur-Yvette, France; CEMHTI - UPR3079 CNRS, Site Haute Température, 1D avenue de la Recherche Scientifique, 45071 Orléans Cedex 2, France; Institut des Matériaux Jean Rouxel (IMN), Université de Nantes, CNRS, 2 rue de la Houssinière, BP32229, 44322 Nantes Cedex 3, France

**Keywords:** Disordered compounds, Substitution, Vacancies, Quantum calculations, Combinatorics

## Abstract

**Background:**

Disordered compounds are crucially important for fundamental science and industrial applications. Yet most available methods to explore solid-state material properties require ideal periodicity, which, strictly speaking, does not exist in this type of materials. The supercell approximation is a way to imply periodicity to disordered systems while preserving “disordered” properties at the local level. Although this approach is very common, most of the reported research still uses supercells that are constructed “by hand” and ad-hoc.

**Results:**

This paper describes a software named *supercell*, which has been designed to facilitate the construction of structural models for the description of vacancy or substitution defects in otherwise periodically-ordered (crystalline) materials. The presented software allows to apply the supercell approximation systematically with an all-in-one implementation of algorithms for structure manipulation, supercell generation, permutations of atoms and vacancies, charge balancing, detecting symmetry-equivalent structures, Coulomb energy calculations and sampling output configurations. The mathematical and physical backgrounds of the program are presented, along with an explanation of the main algorithms and relevant technical details of their implementation. Practical applications of the program to different types of solid-state materials are given to illustrate some of its potential fields of application. Comparisons of the various algorithms implemented within *supercell* with similar solutions are presented where possible.

**Conclusions:**

The all-in-one approach to process point disordered structures, powerful command line interface, excellent performance, flexibility and GNU GPL license make the *supercell* program a versatile set of tools for disordered structures manipulations.

**Electronic supplementary material:**

The online version of this article (doi:10.1186/s13321-016-0129-3) contains supplementary material, which is available to authorized users.

## Background

The structure and properties of disordered condensed systems have always attracted the attention of scientists and engineers [1]. Many unique properties of solid-state materials appear only in a disordered and/or defected state. Of the various types of disorder that exist at different length scales in real compounds [2, 3], local atomic impurities, substitutions and/or vacancies are among the most important. Such point defects are in particular responsible for the unique properties of a number of semiconductors, high temperature superconductors, metallic alloys, ceramics (including piezoelectric), zeolite catalysts and many other types of technologically important materials.

A wide range of experimental techniques exist to explore the local disorder in otherwise crystalline solids, complementary to diffraction techniques which provide information on the average long-range structure, including in favorable cases fractional occupancies an/or mixed atomic compositions on the different crystallographic sites. Because they do not rely on long-range atomic periodicity, local spectroscopies such as solid-state nuclear magnetic resonance (NMR) [4], Raman [5], infra-red or X-ray adsorption near-edge structure (XANES) are particularly relevant to reveal and characterize point defects to then understand how they affect the materials properties [6, 7]. Solid-state NMR, in particular, has successfully been used, often in combination with density functional theory (DFT) calculations of NMR parameters [4, 6, 8, 9], to unravel the effects of substitution disorder in systems as diverse as clays [10–13], layered and microporous silicate catalysts [14–16], ceramics [17, 18], Li-battery [19, 20] and other inorganic oxides [21, 22], chalcogenide semiconductors [23, 24] and doped graphene derivatives [25]. The data that such local probes provide (in the form of chemical shift and electric field gradient tensors or vibrational frequencies, for example) are however often difficult to interpret in terms of local structure around the defected sites. This makes molecular modelling absolutely crucial for the fine understanding of such systems.

The same distinction between local structure and long-range atomic periodicity exists in the theory of solid-state physics, which primarily deals with ideal crystalline systems. One of the most commonly-used approach to connect “periodic” theories with the molecular-level structure probed by local spectroscopies in disordered solids is called the long-range or supercell approximation. The idea is to create a large periodic cell that, within its boundaries, reflects as closely as possible the local structural properties of a disordered system: composition, coordination sequences, etc. This local-level similarity between the real and model (albeit still periodic) systems gives a hope that calculated physical properties will also reflect the real materials properties [26]. While supercells are easy to construct when point defects are in small concentrations and can be considered as isolated, this is more challenging at high defect concentrations, where the relative positions of defects and their interactions become critical. Many factors should be taken into account when implementing the long-range approximation in such systems: the supercell size, its total charge, but also the distribution of local charges, the probability of the supercell configurations, etc.

Different strategies have been described to explore the various atomic supercell configurations that can be generated from a crystal unit cell containing one or several disordered sites (including partially-occupied and/or sites of mixed composition). A particularly successful method called the special quasi-random structures (SQS) [27], consists in constructing one or a small number of supercells with atomic configurations most representative of a random distribution of atom types among the disordered sites. This paper focuses instead on a more systematic and thereby more general strategy, which applies not only to random (as the SQS approach) but also to other types of disorder. It consists in performing a comprehensive search among all possible configurations and thereby offers extended possibilities to explore the local interactions that govern the overall local to long-range compositional (dis)order in such materials. Such exhaustive explorations are however obviously limited by the number of atomic configurations, which very quickly explodes with the number of disordered sites, the concentration of defect atoms and the supercell size. Several excellent theoretical papers [28–30] have therefore described optimized procedures to generate complete sets of unique (i.e., symmetry-independent) configurations, which have been implemented in open [28, 29] and commercial [31] software.

Most of these theoretical developments, however, were initially made for metallic alloys and semi-conductors and have not yet reached certain research areas (and in particular the solid-state NMR community) where molecular modeling is now commonly used to interpret experimental spectroscopic and/or crystallographic data. Possible reasons for this may include the technicality of the corresponding articles [29, 30, 32] and/or discrepancies in the vocabulary employed to describe compositional disorder and its modelling in different communities. In addition, some of the existing implementations suffer from performance issues, limitations on the types of disorder (number of substituents or of disordered crystallographic sites) and/or rather inconvenient input and output formats. It is therefore our opinion that a new software emphasizing the ease of access and use to a broad community of materials scientists, while offering good versatility and performance, was needed.

The new program called *supercell*, which is presented in this paper, aims to fulfill this need. It implements within a single executable multiple tools specifically designed to apply the supercell approximation easily and systematically to a wide range of crystalline solids with compositional disorder. This article describes in very general terms and for a broad audience the basic principles, functionalities, and corresponding algorithms intergrated in the program, and presents its application to a few materials representative of different fields of research where this general approach to the modelling of compositional disorder in crystalline solids could become an essential tool.

## Theoretical aspects

### Supercell approximation

In many types of materials, point defects consist in atomic substitutions or vacancies, meaning that the nature of the atoms occupying one or several crystallographic sites is changed, while preserving the crystallographic positions and hence the overall (average) periodicity of the system. In this case, the disordered structure can be described as a regular periodic structure with well-defined cell parameters, a space-group and a set of crystallographic sites. But contrarily to “ideal” crystals where each crystallographic site is strictly (and fully) occupied by a single type of atom, disordered structures, in their long-range average vision derived from diffraction data, have some of their crystallographic sites partially occupied. This long-range vision, however, has no physical relevance at the local level, where two (or more) atoms cannot of course occupy the same physical position, or one type of atom occupy it partially. What these mixed compositions and partial occupancies mean is that the same crystallographic site in the different periodic images of the original cell can be occupied by different atom types or stay unoccupied (i.e., occupied by a vacancy). A non-periodic occupation of these crystallographic sites will in turn break the periodicity of the system and lead to properties, which, strictly speaking, will depend on the distribution of the different atoms onto these disordered sites in the whole system. In practice, however, many physical properties can be satisfactorily approximated by performing an average (or a summation) over a finite number of small parts of the sample. The essence of the supercell approximation is based on this fact.

Within a supercell numerous atomic configurations can be compatible with the partial occupancies and mixed compositions of the disordered long-range average structure, and the important question is how to explore them efficiently. The approach implemented in our program is an exhaustive exploration where all individual configurations satisfying the conditions are generated and processed. This approach is particularly suitable for small numbers of configurations, but special strategies have also been implemented in the program to treat relatively large cells and/or complex disorder, as will be discussed below.

### Atom combinatorics

The exhaustive search for possible atomic configurations in a given supercell is based on methods of enumerative combinatorics. The problem consisting in distributing atoms among one or several sets of disordered crystallographic sites was reformulated in terms of multinomial permutations.^1^ Let us consider a system with one disordered crystallographic site with multiplicity $K$ and $N$ different types of atoms occupying this site. Vacancies are treated in the same way, as a special “null” atom type. The number of atoms of each type $k_i$, with $i=1\ldots N$, verify $K=\sum _{i=1}^N k_i$. The main two tasks are (1) to calculate the total number of possible permutations and (2) to loop through all of them.

The total number of combinations $P$ can be calculated by a multi-set permutation formula:

1 $$\begin{aligned} P\left( k_1,k_2,\ldots ,k_N\right) =\frac{\left( k_1+k_2+\cdots +k_N\right) !}{k_1!k_2!\ldots k_N!}=\frac{\left( \sum _{i=1}^N k_i\right) !}{\prod _{i=1}^N k_i!} \end{aligned}$$

Equation (1) is a generalization of the well-known binomial distribution formula, obtained for $N=2$ (a typical case being for example the partial occupancy of the considered crystallographic site by one type of atom, the rest being vacancies).

In the (frequent) case where more than one crystallographic site are disordered, the implemented algorithm handles them all at once (unless the user chooses to “freeze” some of them as shown in the program examples), assuming that the permutations within the different sites are independent. The total number of output structures will consequently be $C=\prod _{i=1}^M P_i$, where $P_i$ is number of possible permutations on site *i*, and *M* the total number of independent disordered sites.

## Algorithm implementations in the supercell program

**Fig. 1:**
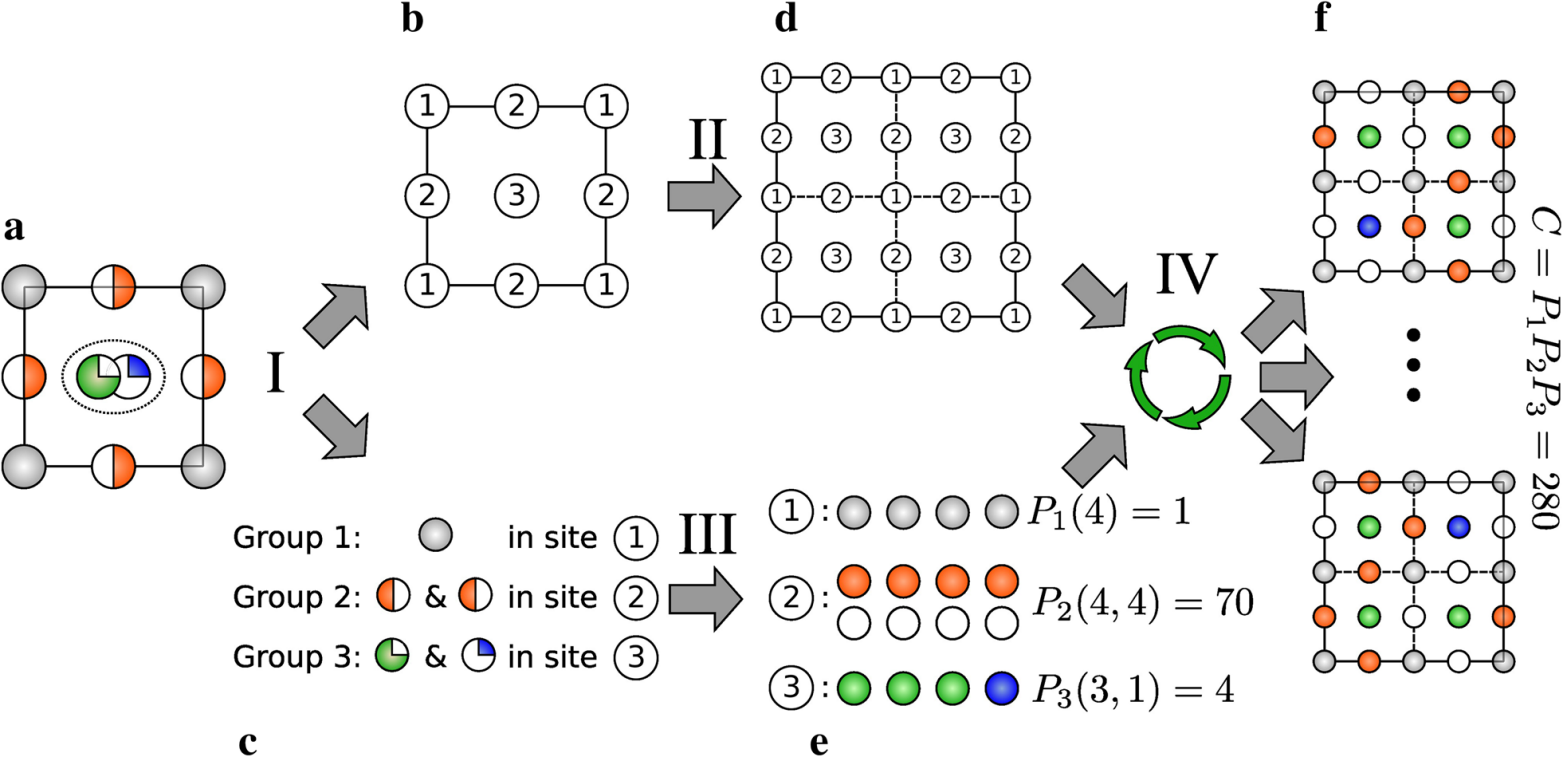
Illustration on a hypothetical disordered 2D crystal of the main concepts and tasks of the supercell algorithm workflow. **a** Input structure consisting of crystallographic positions and occupation values for each atom type, as typically defined in a cif file. **b** Crystallographic sites are sorted into groups 1, 2, and 3. **c** All atom types and corresponding occupancies are then assigned to a group. **d** $2\times 2$ supercell made from cell (**b**). **e** Atoms (in *gray*, *red*, *green* and *blue*) and vacancies (treated as special atoms, in *white*) used for permutations within the groups. **f** Two examples out of many possible resulting periodic structures with full occupancy (or vacancy) of all sites. Stages I–IV are described in detail in text. Numbers of permutations $P_i$ were calculated with formula (1)

The procedure performed by the *supercell* program consists of four main stages that are schematically depicted in Fig. 1.

### Stage I: Assigning crystallographic sites and atoms to groups

First of all, all sites and atoms in the initial structure (Fig. 1a) are sorted out to different groups. Those groups are constructed such that permutation theory can be applied independently to each group. Each object of this type consists of a set of positions and a set of atoms which can occupy all these positions with equal probability and without any restriction. The atom types and crystallographic sites are sorted to groups according to the following rules:

1. each atom type should be assigned strictly to one group,
2. each group should be associated with at least one atom type,
3. each crystallographic position belongs strictly to one group,
4. a group should be associated with at least one position,
5. all atom types within a group occupy all positions assigned to the group with the same probability, and
6. each position cannot be occupied by more than one atom.

In most cases it is enough to treat a group like a crystallographic site occupied by one or more element(s) and/or a vacancy with some population. Three different groups are presented in the example on Fig. 1. Group 1 consists of one crystallographic site, marked as “1” (Fig. 1b), and one atom type: “gray” (Fig. 1c) with 100 % occupancy. The same characteristics apply of course to all images of this site, whether by cell symmetry or periodicity. Group 2 consists of 1 crystallographic site, marked as “2”, and one atom type “orange” occupying 50 % of the corresponding positions, while leaving 50 % vacancies on this site. Group 3 illustrates a more unusual but nevertheless plausible case. This group consists of two distinct but close crystallographic positions, each occupied by a distinct atom type: “green” and “blue”, respectively with occupancies of 75 and 25 %. Because the two distinct crystallographic positions in group 3 are too close in space to be possibly occupied at the same time (a distance criterion that defaults to 0.75 Å, but can be modified by the user), the program reduces them to one single position, marked as “3” in Fig. 1b, and corresponding to the median point between them. This description in terms of groups serves as the basis for the application of the permutation theory.

The *supercell* program implementation assumes that atoms types (represented by different colors in Fig. 1) are separated into different atom labels obtained from the input cif file. Differently-labelled atoms will be treated like different permutation species even though they denote the same element and/or are located in positions that are related by symmetry (in the latter case the program will break the symmetry and assign the atoms to different groups).

### Stage II: Supercell generation

A new (super)cell is produced by replication of the initial one (Fig. 1b) over cell vectors ($\vec {a},\,\vec {b},\,\vec {c}$).^2^ The new cell (Fig. 1d) will have a size of ($l \vec {a},\,m \vec {b},\,n \vec {c}$), where $l,\,m$ and $n$ are natural (positive integer) numbers. Each crystallographic site *p* in the initial cell with Cartesian coordinates $\vec {q}_p$ will have a total of $l \cdot m \cdot n$ images in the supercell with Cartesian coordinates $\vec {q}_p^{i,j,k}= \vec {q}_p + i \vec {a} + j \vec {b} + k \vec {c}$, where $i=0,1\ldots (l-1),\,j=0,1\ldots (m-1)$ and $k=0,1\ldots (n-1)$. It is important to keep in mind that this supercell expansion approach is a special case: the simplest one. It does not allow for example transformations of a primitive cell into a conventional (super)cell, or the opposite. A more general approach exists [33], which creates supercell vectors on the basis of linear combinations of the initial cell vectors, a desirable improvement that will be considered for a future version of the program.

The choice of the $l,\,m$ and *n* supercell-expansion factors are strongly dependent on the initial cell shape, on the targeted properties and (of course) on the computational cost of the calculations needed to predict them. For three-dimensional solids it is often desirable to describe the effects of local disorder to the longest-possible range in all directions of space (or parallel to the cleavage plane for 2D systems). This typically requires that $l,\,m$ and $n$ values are inversely proportional to their respective initial cell parameters to maximize the shortest distance between periodic images in all directions and hence minimize finite-size effects. On the contrary, in other cases where a long-range effect in one direction may be expected, it can be preferable to build instead largely anisotropic supercells [34]. Finally, while properties such as the density of states may often be satisfactorily computed with small supercells [35], other properties like Raman require very large ones.

### Stage III: Occupancy correction

Within the supercell approximation, the composition of the supercell should be as consistent as possible with the composition determined experimentally for the system under study. The third stage of the procedure implemented in our program involves some algorithms that have been specifically designed to help the user obtain the desired composition. This step consists in transforming the occupancy values of all atoms (represented by their labels) in every group into integer numbers of atoms distributed among all allowed positions in the group (Fig. 1e). In the supercell, these positions include all images of the sites assigned to the group by symmetry and by translation of the original cell. This transformation is a classical task of integer programming. The implemented algorithm minimizes the $\chi ^2$ defined below with some restrictions.

2 $$\begin{aligned} \chi ^2=\sum _{i \in G}\sum _{j \in L_i }{\left( R_i^j-\frac{N_i^j}{S_i}\right) ^2} \end{aligned}$$

In this equation the first sum (*i* indexes) runs over all groups ($G$), and the second ($j$ indexes) over all atom labels ($L_i$) associated with group $i$. $N_i^j$ are variables (numbers of atoms) used to minimize (2), with $R_i^j$ the occupancy value provided in the input file^3^ and $S_i$ the number of allowed positions in group *i*. A first restriction is that the sum of all atom occupancies in a group should be less than or equal to the number of possible positions in the group: $\sum _jN_i^j \le S_i$. The second restriction comes from charge balancing. If the corresponding option is switched on (“-c yes”) the combinations of $N_i^j$ which do not satisfy the system charge-balance condition $\sum _{i, j} q_i^j N_i^j=0$ will be discarded. One more restriction is used for fully-occupied groups, i.e., groups *i* such that $\sum _j{R_i^j} = 1$. In such cases the group should remain fully occupied after the procedure, which translates to imposing that $\sum _j{N_i^j} = S_i$. Finally, it is possible to set manually some of the $N_i^j$ values (with the “−p” option, see the examples for details), which will then remain fixed during the minimization procedure. The minimization algorithm implemented in the *supercell* program is quite simple. It goes through all possible values of $N_i^j$, calculating the $\chi ^2$ value and discarding combinations of $N_i^j$ that do not fulfil the restriction conditions. The set of $N_i^j$ values that minimizes $\chi ^2$ will be kept for the next step.

### Stage IV: Processing and storing result structures

Once the occupancy number of all sites has been determined, supercell structures with real atoms and vacancies (rather than partially-occupied or mixed compositions) may be constructed accordingly (Fig. 1f). As mentioned above, the permutation proceeds for all groups independently, which results in a set of structures representing all possible combinations of all groups. Some atom groups can be excluded from this procedure manually, in which case the generated structures will retain partial occupancies for all crystallographic sites within these groups.^4^

As mentioned above, the number of possible combinations may quickly become very large, to the point where storing and analyzing the results can be problematic. Different strategies have been implemented in the *supercell* program to overcome this problem. The program offers different ways to process the output structures before storing them. First of all (this is the default), the structures can be stored directly as is. Secondly, it is strongly advisable to merge structures that are equivalent by symmetry or by translation (using the “−m” option), which tends to considerably reduce the number of structures (depending on the symmetry of the system). Thirdly, Coulomb energy calculations can additionally be performed (“−q” option) to sort out structures on the basis of their (crudely-approximated) energies. This ranking may then be used to sample the resulting structures according to different criteria (“−n” option), which proves a particularly useful tool when the number of distinct structures is high. The number *N* of combinations to store may be picked in any of the following ways (possibly at the same time): randomly, the first and/or last *N* combinations generated, and/or the *N* combinations with lowest or highest Coulomb energy. The sampling algorithm runs after the Coulomb energy calculations and merging algorithm and requires extra memory to temporarily store the sampled combinations.

#### Merging equivalent structures

The purpose of this algorithm is to identify and offer the possibility to store only unique structures, i.e., that cannot be converted to each other by affine transformation. This is a truly central aspect to the treatment of atomic disorder in crystals because, as will be illustrated in various examples discussed below, the total number of combinations can be reduced by up to three orders of magnitude using this procedure, depending on the symmetry of the system. Efficient approaches to perform this task have been found as a result of intense research efforts by several groups in the past [28–30], which largely benefited to the *supercell* program. The method implemented here is based on the use of symmetries, similar in essence to the approach discussed by Hart and Forcade [29] and consists of two stages. During the first stage all possible symmetry operations that apply to the supercell are identified. The second stage is a part of the permutation iteration loop. During this stage, the symmetries calculated before are applied to the current atom configuration.

The symmetry-search algorithm (stage one) generates symmetries on-the-fly and does not use any symmetry information from the input structure^5^ or symmetry databases (no information about space group or Bravais lattice). The symmetry-search algorithm can be split in two parts. First, the algorithm searches for all possible crystallographic point groups for the lattice, to identify all linear transformations (rotations, inversions...etc), which transform the cell to itself. This step is performed on a supercell from which all disordered sites whose distribution is under investigation (i.e., those not fixed by the user) have been removed. This leads to a maximum number of potential symmetry operations which may or may not apply to each individual atomic configuration, once atoms and vacancies are distributed onto these disordered sites. In a second step, the symmetries identified are applied to all atomic positions in the supercell, to then search for translation symmetries (i.e., shift vectors).

Although this approach is not compatible with the “standard space groups notation” it has clear advantages. The first and most important advantage is that the program can be used sequentially: the output cif files of one *supercell* run in which some of the groups have been fixed (hence retaining partial occupancies) can be used as inputs to another run. This possibility greatly extends the applicability as well as the potentialities of the program for large, highly-symmetric and/or complicated structures containing several disordered sites (a principle that is exploited in some of the examples discussed in Additional file 1). Another advantage of this approach is that non-standard cells can be used (including for example structures that have been edited manually).

During the permutation loop the obtained symmetry operations are applied to each permutation. Lexicographic order of permutations allows to combine symmetry-equivalent structures with a run time that scales linearly with the total number of permutations [36], as also used in the implementation by Hart and Forcade [29] (readers are encouraged to look therein for a clear illustration of this principle). A weight parameter reflecting the number of non-unique structures merged together is finally attributed to each unique structure.

#### Coulomb energy calculations

The Coulomb energy calculations implemented in the *supercell* program uses the Ewald summation algorithm, which achieves considerably increased accuracy as compared to a simple truncation for long-range potentials with $1/r$ asymptotic behavior. This algorithm has been described in many sources, and our implementation is based on equations published in [37], with a relative precision of the energy calculations set to $10^{-7}$. The result of our implementation of the Ewald sum was tested by comparing the calculated energies with the Ewald energies obtained with the GULP code [38], which they match perfectly.

### Technical details

The *supercell* program is written in standard C++ language and should be compatible with all popular compilers (MSVC, GNU, Intel Compiler, MinGW, Clang), operating systems (Windows, Mac OS X, Linux, BSD, Windows/Cygwin) and platforms. The version of the program, presented in the paper successfully compiled on Linux platforms with GNU C++ 4.x, Clang 3.4 and Intel^®^ 14.0 compilers. We expect fewer risks of facing technical problems during installation procedures performed with GNU compilers. The CMake configure system is used for dependency check and cross-platform build. The code uses the Boost library for command-line argument processing, advanced file operations and REgular EXpression (regex) parsing. All operations on the structure of the compound (read/write CIF file, structure exploration and modification) are done with a customized version of the OpenBabel [39] library, which was modified by KO to handle partial occupancy in CIF file reading and writing operations. This version of OpenBabel is available from the official Website [40], and the corresponding changes will be merged to the official OpenBabel repository [41]. The *supercell* program also uses the *libarchive* library to archive output structures on-the-fly.

A manual of the program is available in the distribution as a LaTeX source. The manual can be compiled to md (Unix man) and pdf file. Compiling to Unix man is carried out with a modified version of the *latex2man* script [42] (distributed with the program). This script requires Perl to be installed. To compile the manual to PDF, the *pdflatex* program should be available with *latex2man* style files.

Some limitations of the code should be taken into account. The program currently only has a serial implementation and consequently does not exploit the advantages of multiprocessor machines. The implementation of the different algorithms is nevertheless very efficient in terms of CPU and memory load. It can work satisfactorily even on netbooks for all examples discussed in this article. The maximum number of permutations should not exceed a limit of 800 million, above which the program will return an error. Importantly, systems with a total number of combinations well above this limit can still be treated provided that the number of permutations within each group is below the limit. As mentioned above, the program can indeed be executed step-by-step with some groups excluded from permutation. Large supercells can cause the program to crash with arithmetic overflow when the rapidly-increasing number of combinations exceeds $2^{63} \approx 10^{18}$, due to the finite size of integer variables.

At the moment the supercell program distributed as a source code only. It is freely available from https://github.com/orex/supercell. Users are very welcome to submit bugs, features requests, code improvements, examples etc. Snapshot of supercell program can also be found in Additional file 2 (supercell.tar).

### License

The *supercell* code is available for everybody without restrictions, which gives a possibility for all users to check the code, improve it and customize the program for their needs, respecting the license. We kindly ask users to keep in touch with authors to help them improve the program.

## Comparison with existing solutions

As already mentioned above, several solutions exist to systematically explore all possible atomic configurations from the long-range average structure of disordered crystals, which are listed in Table 1. Although all of them share the same general purposes, their implementations, sets of related algorithms and possible applications are quite different.

**Table 1:** Comparisons of programs performing combinatorial treatments of disorder in crystals

	*supercell*	CRYSTAL	SOD	*enumlib*	*eumlib + pymathgen*
Public release	2016	2014	2007	2008	2013
License and availability	GPL, open access	Commercial, free demo version available	GPL, on demand	MIT, open access	
Programming language	C++	Fortran	Fortran	Fortran	Fortran/Python
Interface	Command line interface (CLI)	Custom configuration file			Python script
Input from standard structure files	Yes	No	No	No	Yes
Preprocessing algorithms	Grouping, occupancy correction	–	–	–	(Any)^a^
Non-diagonal supercell expansion matrix	No	Yes	No	Yes	
Multinominal distribution	Yes	No	No	Yes	
Disorder on several independent sites supported	Yes	No	No	Yes	
Random sampling	Yes	Yes	No	No	No
Coulomb energy sampling	Yes	No	No	No	No^b^
Interface to calculations programs	External^c^	Internal, CRYSTAL only	Internal, VASP, GULP	Internal VASP	Internal VASP, GULP
Performance $\hbox {Sn}_{0.5}\hbox {Pb}_{0.5}\hbox {Te}$ $2\times 2\times 2$)^d^	27 min	15 s^e^	N/A^f^	29 h	

$^\mathrm{a}$ Pymathgen supports a wide range of structure manipulation procedures [43]

$^\mathrm{b}$ Coulomb energy sampling and merge algorithm are mutually exclusive within this framework

$^\mathrm{c}$ Input for most calculation programs can be prepared with shell scripts and cif2cell or OpenBabel

$^\mathrm{d}$  The reported time is a dry-run time on Intel^®^ Xeon^®^ X5550 processor. All time-consuming I/O operations were disabled. The example is particularly challenging because the number of symmetry operations (1536) is really high (the same number of permutations on systems of lower symmetry should be processed faster)

$^\mathrm{e}$ The reported duration corresponds to the calculation of the total number of unique structures calculation. The sampling algorithm crashed

$^\mathrm{f}$ The program crashed with memory error. The expected run time is more than a year

The Site-Occupancy Disorder (SOD) program was historically first to implement such functionalities [28]. Since 2007, the code has been used in studies of several classes of disordered solid-state compounds. The *enumlib* program [29] was introduced in 2008 and primarily used thereafter for the treatment of compositional disorder in metallic alloys. It was later (2013) wrapped in the *pymatgen* library [43], which considerably extended the possibilities of the program, allowing to customize the input and output of the program, and offering a variety of tools for data analysis. The commercial CRYSTAL program [31], a well-known solution for DFT calculations, was very recently extended with a part dedicated to the treatment of disorder in crystals [30] (in version CRYSTAL14).

The command line interface (CLI) was chosen for *supercell* to create a user-friendly and ready-to-use, but also flexible program, rather than the custom input-file interface used in SOD, CRYSTAL14 and *enumlib* codes. The CLI is suitable both for manual user input and scripting automation and, combined with a verbose output, allows to set output data properties easily and step-by-step. The *pymatgen* library offers a very powerful Python-script approach to use *enumlib*, which is extremely flexible, but requires at least a basic level of programming skills as well as knowledge of the *pymatgen* library structure and common templates. We note that the functionalities offered by *pymatgen* can also easily be used with our program. As most other solutions the *supercell* program is distributed with a manual (CLI parameters manual: Unix man) and a set of application examples illustrating all features of the program, which are described in detail below (and in Additional file 1).

The functionalities offered by the different programs also present several significant differences. Much attention has been paid in *supercell* to facilitate the initial structure processing steps: direct input from cif files, the grouping functionality, and a flexible occupancy correction procedure. Other programs require that users perform these operations manually (CRYSTAL14, SOD, *enumlib*) or create a Python script for this (*pymatgen*). Contrarily to other programs *supercell* is not distributed with extra tools for output structures analyses and conversion, and authors recommend to use external tools like OpenBabel [39] and cif2cell [44] for the preparation of DFT calculation inputs and fpNMR [45] for structure analyses.^6^ Finally, in contrast with SOD and CRYSTAL14 codes, which are limited to compounds having only one disordered crystallographic site involving two atom types (or one atom type and vacancies), *Supercell*, like *enumlib*, can handle multiple sites with complex compositions.

Some features of the *supercell* code have been developed specifically to facilitate the treatment of the (very frequent) cases where large numbers of permutations are encountered (typically $10^{5}$–$10^{9}$). The code was tested for $\hbox {Sn}_{0.5}\hbox {Pb}_{0.5}\hbox {Te}$ with a supercell consisting of $2\times 2\times 2$ conventional cells. Both SOD and CRYSTAL code crashed during the run, although the CRYSTAL14 program showed excellent performance as far as the calculation of the total number of unique structures is concerned. *Supercell* was 60 times faster than *enumlib* on this particular example. These results suggest that, at least to the best of our knowledge, the *supercell* program is currently the best solution for cases with a large number of permutations.

## Exploring supercell configurations: *supercell* application examples

Distinct configurations generated with a given supercell size are expected to give rise to different calculated properties, but their numbers will often be too large to make systematic calculations practical. The crucial question for many research problems is therefore: how to select the configuration(s) that are most representative of the real system or/and how to average calculated properties from the set of calculated configurations? This question stretches far beyond the choice of the combinatorial approach and the answer will strongly depend on the type disorder present in the materials of interest. The next section illustrates the possibilities and limitations, advantages and disadvantages of the of *supercell* approach through examples based on data published in the literature, and representative of important types of compositional disorder in crystals. More examples can be found in Additional file 1.

### Solid solutions: atomic substitutions in semiconductors

**Fig. 2:**
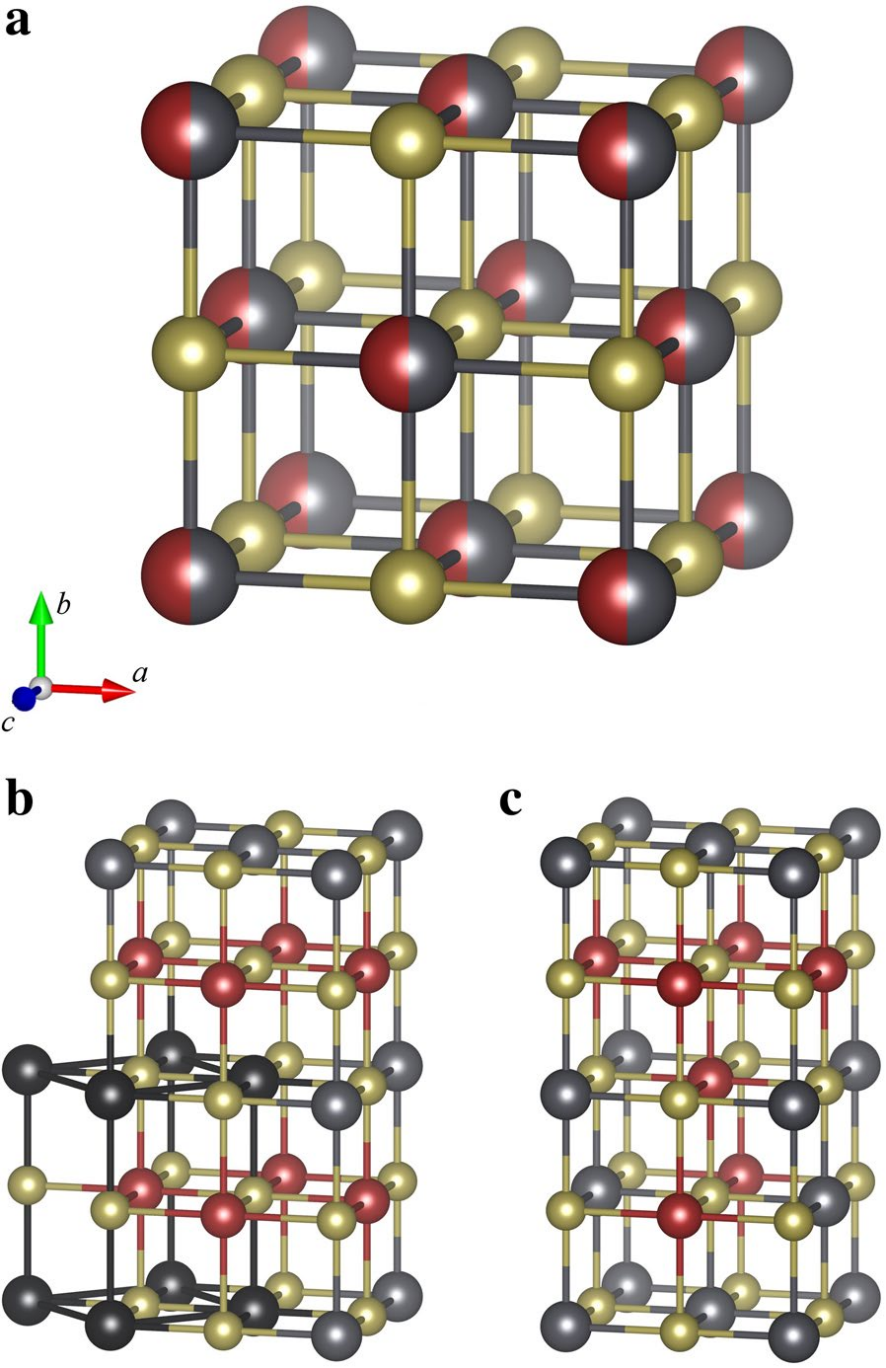
Cubic crystal structure of $\hbox {Sn}_\mathrm{x} \hbox {Pb}_{1-\mathrm{x}}\hbox {Te}$  [60]. **a** original unit cell with space group $\hbox {Fm}\bar{3}\hbox {m}$ (225), and $a = b = c = 6.32\text {-}6.46$ Å. The structure consists of mixed lead and tin (in *dark gray* and *dark red*, respectively) sites and tellurium sites (*brass-colour*) occupying 4a (0, 0, 0) and 4b $\left(\frac{1}{2},\frac{1}{2},\frac{1}{2}\right)$ positions with the same point symmetry $\hbox {m}\bar{3}\hbox {m}$. **b**, **c** Two out of 8 possible unique structures for $x=\frac{1}{2}$ and cell $1\times 2\times 1$ (see Table 2). **b** The highest-symmetry structure corresponds to tetragonal ${\text{P}}4/{\text{mmm}}$ space group (123), whose corresponding primitive cell is highlighted with *black lines*. **c** Lowest-symmetry structure with orthorhombic ${\text{Pmm}}2$ space group (25)

The atomic impurities and substitutions in semiconductors are crucial for building semiconductor electronic devices, lasers, thermoelectric materials etc. A typical example is the narrow-band semiconductor PbTe, which, when doped with tin, is used as an infra-red detector [46, 47]. The crystal structure of this compound is shown on Fig. 2. This IV–VI rocksalt semiconductor alloy system has a NaCl structure type, with typically fully-random atomic substitutions taking place at the 4b position. Some recent studies used an ab initio approach to investigate the band-structure properties of this system as a function of the amount and type of doping atoms, including Ga, In, Tl [48] or Sn [35].

During the alloy formation process, the minimization of the Gibbs energy ($G=H-TS$) of the system involves a competition between the enthalpy $H$, which promotes ordering in the system, and entropy *S*, which drives it towards a fully disordered state. The result of this process depends on the alloy composition. It is important in this context to be able to generate both ordered and disordered configurations, and the *supercell* program can be a valuable tool for this purpose. In the particular case of $\hbox {Sn}_\mathrm{x}\hbox {Pb}_{1-\mathrm{x}}\hbox {Te}$ compounds, the program was used with supercell sizes up to $2\times 2\times 2$ (based on the conventional cell containing 8 atoms) and various concentrations of dopants (Table 2). These concentrations and supercell sizes were taken to reflect the structures calculated in Refs. [35, 48]. The symmetry-merging algorithm significantly reduces in all cases the number of configurations to treat. For most concentrations and supercell sizes presented in the table, it is feasible to conduct electronic-level calculations (i.e., DFT) of the desired properties for all atomic configurations. But in some cases such as, for instance, the $2\times 2\times 2$ supercell with $x=\frac{1}{2}$, the number of configurations is too high (larger than $4\times 10^5$) for this, and sampling strategies must consequently be employed.

**Table 2:** Total number of possible atom combinations for different substitution levels *x* and different supercell sizes of the $\hbox {A}_\mathrm{x} \hbox {Pb}_\mathrm{1-x}\hbox {Te}$ system

*N*	8	16	24	32	64
Cell, $a\times b \times c$	$1\times 1 \times 1$	$1\times 1 \times 2$	$1\times 1 \times 3$	$1\times 2 \times 2$	$2\times 2 \times 2$
Symmetry operations	192	128	192	256	1536
$x=\frac{1}{16}$	N/A	N/A	N/A	16 (1)	496 (5)
$x=\frac{1}{8}$	N/A	8 (1)	N/A	120 (5)	35,960 (71)
$x=\frac{1}{4}$	4 (1)	28 (4)	220 (9)	1820 (33)	10,518,300 (8043)
$x=\frac{1}{2}$	6 (1)	70 (8)	924 (34)	12,870 (153)	601,080,390 (404,582)

*N* is the total number of atoms in the supercell. The number of unique (non-symmetric) combinations are given in parenthesis. The total number of combinations depends only on *N*, whereas the number of unique combinations can depend on the supercell formula $a\times b \times c$. The number of combinations for substitutions $1-x$ is equal to the number of combinations for $x$ and are consequently not shown

#### Ordered structures

Order-disorder transitions are quite common in solid solutions [49]. A recent study showed in particular that the enthalpy of many type IV–VI solid-solution compounds was lower in the ordered state than in disordered states [50]. Such ordered configurations can easily be obtained with the *supercell* program. This is illustrated here for the supercell $1\times 2\times 1$ of the $\hbox {Sn}_{0.5}\hbox {Pb}_{0.5}\hbox {Te}$ system. As can be seen from Table 2, the total number of symmetry-unique structures for this case is 8. All structures can be easily be generated and processed. Although the *supercell* program does not produce information about spacegroup and/or primitive cell of the structures, the configurations of high and low symmetry can be easily separated on the basis of the weight of the structure (i.e., the number of configurations equivalent by symmetry and/or translation to the considered structure). The number of symmetry operations for configuration *i* can be calculated with the formula $N{/}w_i$, where *N* is the total number of symmetry operations and $w_i$ the weight of structure with index *i*. The two configurations of highest and lowest symmetry obtained with the supercell $1\times 2\times 1$ of the $\hbox {Sn}_{0.5}\hbox {Pb}_{0.5}\hbox {Te}$ system are shown on Fig. 2b, c, respectively. They are each characterized by 64 and 4 symmetry operations in the supercell representation, respectively. An analysis of the high-symmetry structure with a crystallographic visualization software indicates that it reduces to tetragonal ${\text{P}}4/{\text{mmm}}$ space group (123) with a 4-atom primitive cell shown in black in Fig. 2b whereas the lowest-symmetry structure corresponds to space group ${\text{Pmm}}2$.

#### Random disorder: special quasirandom structures

The most intuitive approach to model fully-random atom substitution is to randomly pick up a certain number of structures from the full set. This approach, however, is not efficient in the sense that a large number of randomly-selected structures are necessary to reliably predict average properties, which implies a large set of computationally-demanding electronic calculations. Zunger et al. showed that the properties of fully-random alloys can be obtained in a considerably more efficient manner by constructing non-random configurations called “special quasirandom structures” (SQS) [27]. SQS are special configurations that, for a certain supercell size, reproduce as closely as possible a set of close-range radial correlation functions (a simplified definition is given below) of the wholly-random system.

The combination of the *Supercell* program with a structure-analysis tool can be used to calculate the *k*-atom correlation functions (where *k* is typically limited to 2 or 3-atom interactions), to then identify the configurations that satisfy the SQS criteria. An implementation of this method for the $\hbox {Sn}_{0.5}\hbox {Pb}_{0.5}\hbox {Te}$ system is provided as an example in the *supercell* program distribution. In this simple example, which has a single disordered site with mixed 50 %/50 % Sn/Pb composition, these correlation functions may be described with the following formulas, which are a simplification of the general theory for binary alloys considering two-atom correlations only. We first introduce the parameter $S_i$ (with *i* the atom index), which takes the value +1 when *i* is an atom of type A and −1 for an atom of type B. In this case, the atom-pair correlation function can be calculated as:

3 $$\begin{aligned} \bar{\Pi }_{2,m} = \sum _{i,j \in r_{ij}=R_m } S_iS_j \end{aligned}$$

where the first index in $\bar{\Pi }$ means that it is a two-atom correlation, the second index enumerates the coordination spheres of atoms, $R_m$ is the radius of *m*th correlation sphere and $r_{ij}$ is the distance between atoms *i* and *j*. The summation goes over all pairs *i*, *j* with distance between atoms equal to $R_m$ (which takes discrete values in the average crystal system). Generally, the correlation functions for fully-random binary alloys can be calculated analytically with the formula $\bar{\Pi }_{k,m}=(2x-1)^k$, where $x=\frac{N_A}{N_A+N_B}$ is the substitution rate. For the special case of a fully-random alloy where $x=0.5$ (equal numbers of A and B atoms), these correlation functions all reduce to zero. So the configurations matching the SQS criteria in this case should have null correlation functions in their first *n* coordination spheres, where the number *n* should be as high as possible. Out of the 153 distinct configurations identified for supercell size $1\times 2\times 2$, our analysis of the $\hbox {Sn}_{0.5}\hbox {Pb}_{0.5}\hbox {Te}$ system found 6 structures with null correlation coefficients for the first 3 shells (ignoring Te atoms), which means that their local Pb/Sn arrangements perfectly mimic those of a randomly-disordered system for cation-cation distances up to $\approx$8 Å.

The proposed approach calculates correlation functions for all possible configurations and is therefore technically limited to small structures.^7^ Traditionally, researchers use the alloy-theoretic automated toolkit (ATAT) package [51] to work with random alloys. The package is highly oriented towards this type of disordered systems and offers many useful functionalities such as the cluster expansion [52], SQS structure generation (both “brute-force” and stochastic algorithms) and interface to ab initio codes. The supercell program targets a broader range of systems, which potentially includes but is not limited to the randomly-disordered systems targeted by the SQS approach, and any type of target correlation function could be considered to favor or penalize instead some chosen interactions (e.g., A-A first-neighbor interactions in the simple two-atom example discussed above). The exhaustive searches performed by supercell and the set tools that is contains are particularly adapted to the treatment of non-random types of disorder, which are the focus of the next two sections.

### Correlated disorder: ice $\hbox {I}_\mathrm{h}$

**Fig. 3:**
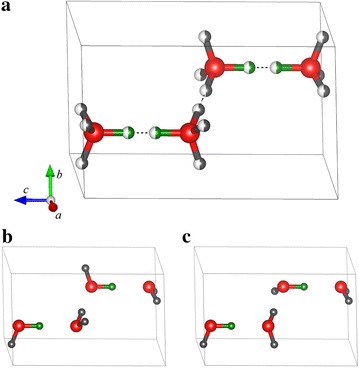
Crystal structure of ice $\hbox {I}_\mathrm{h}$ [61] with correlated disorder. **a** original unit cell with space group ${\hbox {P}{6}_3/mmc}$ (194), $a\, = \,b\, =\, 4.497479$ Å and $c = 7.322382$ Å $(\alpha = \beta = 90^{\circ }, \gamma = 120^{\circ }$). All O atoms (*red*) are on fully-occupied 4f site. H atoms have two positions: H1 4f (*green*) and H2 12k (*gray*), both with 50 % occupancy (see text). *Dashed line* shows unrealistic H–H distance of $\approx$0.8 Å. In **b** and **c** are two configurations satisfying the restrictions on H atoms positions that result in correlated disorder in this system, with space groups $\hbox {Cmc2}_1$ (36) and **c** ${\hbox{Cc}}$ (9), respectively

In some disordered systems the sitings of atoms onto the different disordered crystallographic sites are not independent from each other, but follow instead some restrictions. Correlated disorder appears when the restrictions impose a long-range correlation in atom locations, i.e., when the placement of one atom or a vacancy imposes or prevents the placement of another at a nearby position, which in turns affects the next position, etc. A well-known example of such type of disorder is the common form of ice ($\hbox {I}_\mathrm{h}$). Many specific properties of ice, including its residual entropy, large static dielectric permittivity, electrical polarizability and conductivity can be attributed to proton disorder [53]. The crystal structure of ice $\hbox {I}_\mathrm{h}$ (as given in the cif file) is shown on Fig. 3. The system has a hexagonal lattice with a well-defined oxygen position in site 4f. Hydrogen atoms, on the other hand, occupy two distinct positions, each with 50 % probability: H1 (4f) and H2 (12k). The placement of hydrogen atoms should obey two rules. First, ice being a crystal made of water molecules, the number of H atoms in the first coordination sphere of every O atom should always be two. Second, the placement of a H atom depends on the presence (or absence) of another one on the close-by H site, whereby H–H close contact (dashed line in Fig. 3) is avoided. The disorder in this system is therefore clearly correlated: the placement of one H atom directly affects the placement of all other H atoms around it, which in turn affects the placement of others, etc. The comprehensive exploration of atomic configurations implemented in *supercell* can be used to generate structures that are then selected on the basis of the two criteria described above.

Running the *supercell* program for the initial cell of ice ($\hbox {I}_\mathrm{h}$) yields a total of 5544 combinations, which reduces to 288 distinct configurations upon application of the symmetry-merging algorithm. The configurations were analyzed with the GULP program [38], which revealed that 30 out of the 288 structures have all their O atoms 2-coordinated, 23 have no H–H close contact, and only 2 structures satisfy both conditions simultaneously. Coulomb energy calculations (using the “−q” option with O and H charges set to −2 and +1, respectively) shows that these 2 structures have (not surprisingly) the lowest Coulomb energy, which illustrates the potential of this criterion for a pre-selection of structures in larger systems with different oxidation states on the substitution sites.

The configuration-generation step becomes more complex for cells larger than the initial one. For cell $1\times 2\times 1$, for example, the total number of unique configurations will be 11 million, which is too large to process in the same way as the initial cell (this also applies of course to $2\times 1\times 1$ and $1\times 1\times 2$ supercells, but because the initial c parameter is larger than parameters a and b, the latter would be less interesting because of its strong anisotropy). The systematic structure-analysis approach (using GULP) described above reaches its limit considerably more quickly than the *supercell* program, which is able to generate ice $\hbox {I}_\mathrm{h}$ configurations for supercells up to size $2\times 2\times 1$ ($\approx$ 4.2 $\times 10^{17}$ combinations). This was nevertheless achieved, using a quite specific procedure that requires advanced understanding of combinatorics and of the algorithm implementation within the *supercell* program, and is consequently presented in Additional file 1. The results reveal that only 9 out of a total number of 11 million distinct $1\times 2\times 1$- (or $2\times 1\times 1$-) supercell configurations satisfy both the H–H avoidance and the presence of H$_{2}$O molecules only (see Additional file 1 for details).

The necessity to keep only configurations containing H$_{2}$O molecules is furthermore representative of another type of disorder that may be found in many molecular crystals or in systems consisting of guest molecules confined within a crystalline host matrix. The program should ideally be able to retain (or even better to explore) only the configurations where certain specified molecular fragments would be present. This is done as an external post-processing step in the $\hbox {I}_\mathrm{h}$ example, with a procedure that may easily be extended to other systems. While this is an appealing functionality to possibly include in future developments of the *supercell* program, it should be done without loss of generality or ease-to-use, which makes it very challenging (and far beyond the scope of the present work).

### Short-range order: the Loewenstein’s rule in aluminosilicates

The disorder in many materials is neither random nor correlated. This intermediate case is characterized by large amounts of possible stable configurations, as in randomly-disordered crystalline systems. From another side, however, the difference in energy between different configurations is significant, as in correlated disordered materials, but to a smaller extent. The main difference between this type of compounds and systems with correlated disorder is that many local configurations of higher energy are stable enough to be present in the real system. In ice $\hbox {I}_\mathrm{h}$, in contrast, structures with short H–H distances, which obviously have high energies, will be transformed to one of the low-energy configurations during an energy minimization procedure. Another scenario is the case where a propensity to local ordering (possibly a strict one) applies at the local level (typically two ions strongly repelling each other), but where the concentration of defects is such that this local ordering does not propagate further than a few atomic shells.

A typical example of such “intermediate” disorder is the Al–O–Al avoidance rule (also called the Loewenstein’s rule [54]) between 4-coordinated Al atoms in alumino-silicate materials. The rule dictates that the Si/Al atom substitution in these systems tends to minimize the number of such Al–O–Al bonds, thus minimizing the total energy of the system. In contrast with the correlated disorder of the ice $\hbox {I}_\mathrm{h}$ system discussed above, where violations of the structural restrictions yield unstable and hence forbidden configurations, structures that contain Al–O–Al bonds may very well be stable, albeit energetically unfavorable. And as a matter of fact the Loewenstein’s rule is not strict and is violated in many systems [55]. The amount of violations typically increases with the synthesis temperature, which maximizes the effect of the entropy contribution to the Gibbs free energy, and with the cooling rate, which should be fast enough to limit local atomic rearrangements in the favor of Si–O–Al bonds.

**Fig. 4:**
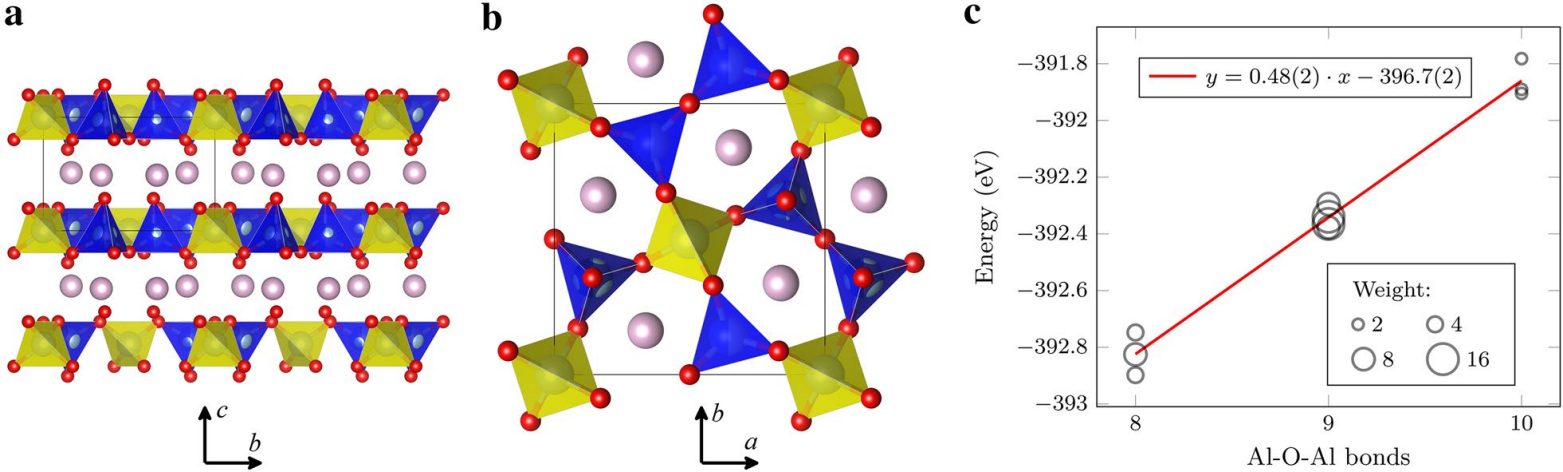
Crystal structure of Ca_2_Al_2_SiO_7_ viewed **a** from the side and **b** from above layers and Al–O–Al bonds energy plot (**b**). **a**, **b** Tetragonal cell with spacegroup $\hbox {P}\bar{4}{2}_\mathrm{1m}$ (113) and *a* = *b* = 7.716 Å and *c* = 5.089 Å. Wyckoff site 2a labelled T1 (*yellow tetrahedra*) is fully occupied by Al atoms and Wyckoff site 4e, labelled T2 (*blue tetrahedra*) is filled with a mix of Al (50 %) and Si (50 %) atoms. **c** Energy of configurations for cell $1\times 1\times 2$ vs the number of Al–O–Al bonds in isolated groups. The slope of linear regression (0.48 eV) agrees well with previously reported value of 0.52 eV [21]

The Si/Al disorder in the gehlenite system Ca$_{2}$Al$_{2}$SiO$_{7}$, for example, was investigated in depth by Florian et al. with a combination of experimental NMR and DFT calculations [21]. Their work beautifully illustrates how a very simple type of disorder (50/50 mixed Al–Si composition on one crystallographic site) results in great extents of complexity at the local level, as probed with local spectroscopic techniques (NMR in this case). Such a complexity requires the combination of many advanced experimental and computational tools to decipher, and is furthermore expected to quickly increase as the number of disordered sites increases. This highlights the essential role of modelling to unravel the molecular-structure-property relationships in such materials. Figure 4a shows the layered structure of Ca$_{2}$Al$_{2}$SiO$_{7}$, which consists of aluminosilicate sheets separated by Ca layers. The Si–Al substitution takes place at tetrahedral positions (T2 sites, shown as blue polyhedrons) forming pairs of two connected tetrahedra that are only connected to pure-Al T1 tetrahedral sites (shown in yellow). Due to this particular arrangement of T1 and T2 sites, the number of Al(T1)–O–Al(T2) connectivities in the system is constant (4 per cell) and the total number of Al–O–Al bonds is entirely determined by the number of Al(T2)–O(2c)–Al(T2) connections, i.e., the numbers of pairs of T2 sites occupied by two Al atoms.

The *supercell* program was applied to a Ca$_{2}$Al$_{2}$SiO$_{7}$ supercell of size $1\times 1\times 2$, which contains 8 positions with mixed Al/Si composition, over four T2–T2 tetrahedron pairs. The total number of possible permutations is $C_8^4=70$, which reduces to 10 distinct configurations. The total number of Al–O–Al bonds within each group varies from 8, when all T2–T2 pairs have one Al and one Si atom, to 10, when two of the pairs are occupied with only Al atoms and the other two with only Si atoms. A geometry optimization procedure (atomic positions only, see “Appendix” for details) was applied to each structure to obtain the total energy as a function of the number of Al–O–Al bonds that the structure contains. The results are presented on Fig. 4c, which shows a strong correlation between the total energy of the structures and the number of Al–O–Al bonds, which provides an estimation of the energetic cost of such bonds, in good quantitative agreement with previously-reported results and with the Loewenstein’s rule [21]. This good agreement was rather unexpected given the exaggeratedly small system chosen here to illustrate how the *supercell* program may be used in such a context. The models used in Ref.  [21] were based on much bigger supercells of size $2\times 2\times 3$ (leading to over $3\times 10^{13}$ configurations before symmetry merging), which were constructed “manually”. It is important to realize that even such a big system could be treated by the *supercell* program, using a procedure that is described in Additional file 1.

## Conclusions

In the present paper we describe the theory and methodology of the supercell approximation for the modelling of crystalline structures with compositional (i.e., chemical) disorder. The new *supercell* program contains an all-in-one implementation of a full set of algorithms that are crucial to conduct this type of analyses, all of which have been described here. It offers a good alternative to other software solutions dedicated to the treatment of disorder in crystals, which provided opportunities to fully test and validate our algorithms’ implementations. The efficient exhaustive search over all possible configurations performed by *supercell* for small- to medium-size systems makes it possible to describe in depth the distributions of local compositions and resulting local geometrical distortions in systems with random as well as non-random types of disorder. The free open-source license of *supercell* as well as its compatibility with any ab initio code via the OpenBabel and Cif2cell package and use of CIF as input and output format, makes it a valuable alternative to the implementation embedded in the commercial CRYSTAL package [31]. We show that it offers significantly improved performance and versatility over the *enumlib* [29] and SOD program [28] (other comparable free open-source solutions) as well as additional tools for the crucial step of structure selection.

The *supercell* program should be treated as a completed software, even though we encourage users to suggest and/or implement improvements that would keep and possibly improve the broadness of its applicability. The released version has well-tested algorithms, a powerful command-line user interface that makes it easily embedded into shell scripts, and support material, including a manual and examples. It can be applied to a wide range of important materials whose properties are crucially impacted by atomic substitutions characterized by different types and extents of local to long-range (dis)order, some of which have been illustrated here (in the main text and/or in Additional file 1). This includes semiconductors and various oxides (including piezoelectric ceramics, see Additional file 1) and chalcogenides, but the list is far longer than this and may include for example ion-conducting materials and many others. Future developments will focus on improved ways to solve the problem of structure selection from large sets of configurations. For the moment this is done solely on the basis of fast and universal Coulomb energy calculations, which for many compounds can give good results [57]. Significant improvement can however be done by total energy calculations of each configuration using classical force fields, which by definition are not universal. This task is therefore very challenging, but would broadly increase the ability to solve complex Materials Science problems based on the *supercell* program. Other ways to improve the program would be to embed the SQS search functionality (which was done with an external program in the $\hbox {Sn}_{0.5}\hbox {Pb}_{0.5}\hbox {Te}$ example discussed here), and to support non-diagonal supercell expansion matrices to explore different supercell shapes.

## Availability and requirements

**Project name:** supercell

**Project home page:**https://github.com/orex/supercell

**Operating system(s):** Linux, can be compiled for other systems.

**Programming language:** C++

**Other requirements:** Boost 1.46 or higher, CMAKE 2.6 or higher, OpenBabel [40, 41], Eigen 3.x, LibArchive (optionally). Perl and LaTeX are needed to compile manual.

**License:** GNU GPL

**Any restrictions to use by non-academics:** according GNU GPL terms

## Appendix: Ca$_{2}$Al$_{2}$SiO$_{2}$ energy calculation details

The geometry optimizations were performed with fixed unit cell parameters using the plane-wave based DFT with pseudopotential implemented in the VASP code [58]. The input parameters were optimized to achieve a maximum performance with the required energy precision. The energy cut-off was 500 eV; k-points mesh was $4\times 4\times 3$. Symmetrization was switched off. Ionic positions were optimized using the conjugate gradient method. Convergence threshold criterion was that all forces should be <0.005 eV/Å. We note that the energy of geometry-optimized structures is only an approximation to the total energy, which consists of both configurational (potential) and kinetic energies. More precise results could therefore in principle be obtained by replacing the energy optimizations by molecular dynamic runs (at the DFT level) at the target temperature, but the CPU-time requirements of such calculations make it irrelevant in the context of this work.

## Additional files

10.1186/s13321-016-0129-3 tutorial.pdf --- Short introduction to supercell program.
10.1186/s13321-016-0129-3 supercell.zip --- The snapshot of supercell code from github.

## Footnotes

1 Strictly speaking, permutations and combinations are a special case of multinomial distributions. But further below we will use these terms as synonyms of multinomial distribution sampling.

2 For simplification purposes, the figure illustrates a 2D cell, whereas the text describes the procedure in three dimensions.

3 In this procedure, vacancies are not treated like “special atoms”, so $N_i^j$ are numbers of real atoms in the groups. The number of vacant positions in group *i* can be calculated as $V_i=S_i-\sum _j{N_i^j}$.

4 All sites in each group that the user wants to freeze should be specified as fixed, otherwise the program will return an error.

5 Regular cif files with space group information may of course be provided as the input, but this structure will be converted by OpenBabel library to a structure with *P*1 space group, which our program will use.

6 Although the fpNMR program is strongly oriented towards DFT calculations of NMR parameters, it offers a broad range of general functionalities for structure analyses.

7 For bigger systems is always possible to randomly pick a reasonable number of configurations to calculate the corresponding correlation coefficients, but the probability of finding good candidate SQSs (and even more so to find the best ones) would then be small. In this case it is better to directly construct the SQSs or use stochastic generation approach [56].
