# Supplementary material for: Supercell program: a combinatorial structure-generation approach for the local-level modeling of atomic substitutions and partial occupancies in crystals
Source: J Cheminform. 2016 Mar 31;8:17. doi: 10.1186/s13321-016-0129-3 (PMC4818540; doi:10.1186/s13321-016-0129-3)
Supplement: Supplementary file 1 — 10.1186/s13321-016-0129-3 tutorial.pdf --- Short introduction to supercell program. [file 13321_2016_129_MOESM1_ESM.pdf]

Supercell program tutorial.

Kirill Okhotnikov, Thibault Charpentier and Sylvian Cadars

March 17, 2016

Examples of *supercell* program application.

In the tutorial we discuss technical details of the *supercell* program use. All the examples are based on literature data, which was compared in detail to the results obtained with *supercell* where possible.

Basic functionality: $Ca_2Al_2SiO_7$.

To understand how the *supercell* program works it is necessary to know the structure of Crystallographic Information File (CIF), which used both as the output and input formats. (The conversion to a number of other format is made very easy by the OpenBabel library, which is used by the program). The CIF file is a standard text file format for representing crystallographic information. The format promulgated by the International Union of Crystallography (IUCr) and the detailed information about it can be found at IUCr site (`http://www.iucr.org/`)

The structure of the sections of a CIF file relevant to our program will be illustrated with the `Ca2Al2SiO7.cif` file located in the `supercell/data/example` folder.

```
data_Ca2Al2SiO7
_cell_length_a                   7.716
_cell_length_b                   7.716
_cell_length_c                   5.089
_cell_angle_alpha                90
_cell_angle_beta                 90
_cell_angle_gamma                90
_cell_volume                     302.982
_symmetry_space_group_name_H-M   'P -4 21 m'
_symmetry_int_tables_number      113
loop_
_space_group_symop_operation_xyz
x,y,z
1/2-y,1/2-x,z
y,-x,-z
1/2-x,1/2+y,-z
-x,-y,z
1/2+y,1/2+x,z
-y,x,-z
1/2+x,1/2-y,-z
loop_
_atom_type_symbol
_atom_type_oxidation_number
  Ca1  +2
  AlT1 +3
  AlT2 +3
  SiT2 +4
  O1   -2
  O2   -2
  O3   -2
loop_
_atom_site_label
```

```
33 _atom_site_fract_x
34 _atom_site_fract_y
35 _atom_site_fract_z
36 _atom_site_occupancy
37 AlT1 0.00000 0.00000 0.00000 1.00000
38 AlT2 0.14310 0.35690 0.95280 0.50000
39 SiT2 0.14310 0.35690 0.95280 0.50000
40 O1   0.50000 0.00000 0.18840 1.00000
41 O2   0.14180 0.35820 0.28320 1.00000
42 O3   0.08720 0.17060 0.80330 1.00000
```

The file consists of cell and spacegroup information at lines 2-21 (required by *supercell*), atoms charges (lines 22-30, optional) and atomic positions (lines 32-43, required). The partial occupancies of some sites (last column in lines 32-43) are a feature specific to disordered crystals. In the case presented here, partial occupancies are found for AlT2 and SiT2 sites, which are highlighted above. Both correspond to the same crystallographic position and will therefore be assigned to a single group by the *supercell* program. All other sites are fully occupied and will remain as such throughout the procedure.

The *supercell* program is a user-friendly command-line software. It can be directly applied to cif files. Let us use it for an `Ca2Al2SiO7.cif` file in `supercell/data/examples` folder.

```
supercell -d -i Ca2Al2SiO7.cif -m
```

The output of the command will be

```
kirill@asus-laptop:~/supercell/data/examples$ supercell -d -i Ca2Al2SiO7.cif -m
-----------------------------------------------------
-                Supercell program                  -
-----------------------------------------------------
-       Authors:   * Kirill Okhotnikov              -
-                   (kirill.okhotnikov@gmail.com)   -
-                  * Sylvian Cadars                 -
-                   (sylvian.cadars@cnrs-imn.fr)    -
-----------------------------------------------------

Initial system:
  Chemical Formula: Al4 Ca4 O14 Si2

Supercell system (1x1x1):
  Size a=7.716, b=7.716, c=5.089

Current charge balance option is "try"
Total charge oxidation state (cif):  0
Total charge used:   0

----------------------------------------------------------------
| Atom Label    |        charge           | mult  | occup x mult
|               | Ox. state      | Used   | (cif) |
----------------------------------------------------------------
|  AlT1         |  3             |  3     |  2    |  2
|  AlT2         |  3             |  3     |  4    |  2
|  Ca1          |  2             |  2     |  4    |  4
|  O1           |  -2            |  -2    |  2    |  2
|  O2           |  -2            |  -2    |  4    |  4
|  O3           |  -2            |  -2    |  8    |  8
|  SiT2         |  4             |  4     |  4    |  2
----------------------------------------------------------------

Chemical formula of the supercell: Al4 Ca4 O14 Si2
Total charge of supercell: 0

```

```
37 ---------------------------------------------------
38  Identification of groups of crystallographic sites
39 ---------------------------------------------------
40
41  Group 1 (2 atomic positions in supercell):
42   * Site #1: AlT1 (occ. 1) -> FIXED with occupancy 1.000.
43
44  Group 2 (4 atomic positions in supercell):
45   * Site #1: AlT2 (occ. 0.5) -> distributed over 2 positions out of 4 (actual occ.: 0.500).
46   * Site #2: SiT2 (occ. 0.5) -> distributed over 2 positions out of 4 (actual occ.: 0.500).
47   Number of combinations for the group is 6
48
49  Group 3 (4 atomic positions in supercell):
50   * Site #1: Ca1 (occ. 1) -> FIXED with occupancy 1.000.
51
52  Group 4 (2 atomic positions in supercell):
53   * Site #1: O1 (occ. 1) -> FIXED with occupancy 1.000.
54
55  Group 5 (4 atomic positions in supercell):
56   * Site #1: O2 (occ. 1) -> FIXED with occupancy 1.000.
57
58  Group 6 (8 atomic positions in supercell):
59   * Site #1: O3 (occ. 1) -> FIXED with occupancy 1.000.
60
61 Minimal distance between atoms of two distinct groups: 1.68147 A.
62
63 ------------------------------------------------
64 The total number of combinations is 6
65 ------------------------------------------------
66 8 symmetry operation found for supercell.
67 Combinations after merge: 2
```

The program output gives an information about the initial and supercell system sizes, the total charge, and the chemical formula. The information about atomic species properties in the system are summarized in the table (lines 21-32). The multiplicity parameter is calculated using the symmetry information and atomic positions. The total charge is calculated using `_atom_type_oxidation_number` data in the CIF file or user-defined charges, specified with commands of the type: `-p "Ca1:c=+2" -p "O1:c=-2"`... See the manual and other examples below for more information on the `-p` option. The information about the total number of combinations is presented at line 64. The last two lines (66-67) appear only when the `-m` option is present in the command line.

To generate structures for cell $1 \times 1 \times 2$ (the example presented in the main paper), the *supercell* program should be run with the following parameters:

```
supercell -i Ca2Al2SiO7.cif -s 1x1x2 -m -o Ca2Al2SiO7_cell_1x1x2
```

where the supercell size is controlled by the `-s` option (with "x" letter to represent the "×" symbol) and the output file name prefix by the `-o` parameter.

Sampling methods: $Ca_2Al_2SiO_7$.

The number of combinations increases extremely rapidly with the number of atoms in the supercell (see main text and table 1 for example). In the case of the $Ca_2Al_2SiO_7$ compound, the number of unique combinations for a $2 \times 2 \times 2$ supercell will be 9 402 622 which is too high to process and store, such that sampling is required. *Supercell* offers a few methods of sampling. All of them are controlled by the `-n <type><number>` option, where `<type>` is a letter that determines the sampling method (`r` for random, `l` or `h` for lowest- or highest-E_C structures, `f` or `a` for first or last-generated generated configurations) and `<number>` is the number of sampled configurations. To get 100 configurations picked randomly for the supercell $2 \times 2 \times 2$ the program should be executed with the following parameters:

```
supercell -i Ca2Al2SiO7.cif -s 2x2x2 -m -n r100 -v 2 -o Ca2Al2SiO7_r100
```

The option `-v 2` switches the verbosity to level 2, to follow the program execution, this example being time consuming.

Increasing the supercell size to $2 \times 2 \times 3$ gives a total number of combinations around 3.2×10^{13}. This number is too high to be treated by *supercell* directly, but some of random configurations can nevertheless be created with our program, even for such big system. To perform the operation, the initial file should be processed with *supercell* program first.

```
supercell -i Ca2Al2SiO7.cif -p "*:fixed" && mv supercell_i0.cif Ca2Al2SiO7_abg.cif
```

The parameter `-p "*:fixed"` means, that all of the groups should be fixed (stored to output file with the initial partial occupancies). The result file `Ca2Al2SiO7_abg.cif` will then be used as an initial structure, but with P1 space group. It contains four crystallographic sites with partial Al/Si occupation. The sites `(Al|Si)T2` should be renamed to `(Al|Si)T2a` (for $z = 0.95280$) and `(Al|Si)T2b` for positions with $z = 0.04720$. Because the group identification procedure in the *supercell* program uses the site labels as a first criterion (see main text for details), this new input CIF file now results in the identification of 2 independent groups of sites. The permutations can now be performed step by step, with the commands:

```
rm Ca2Al2SiO7_step*.cif
supercell -i Ca2Al2SiO7_abg.cif -s 2x2x3 -p "*T2b:fixed" -n r1 -o Ca2Al2SiO7_step1
supercell -i Ca2Al2SiO7_step1_ir*.cif -n r1 -o Ca2Al2SiO7_step2
```

The first *supercell* execution generates one random structure (with `-n r1`) within the T2a group, leaving T2b group partially occupied (`-p "*T2b:fixed"`). The next command generates a random configuration within the T2b group, based on the previously-generated file (`Ca2Al2SiO7_step1_ir*.cif`). Strictly speaking, the generated structure is nearly random, not fully. The splitting of T2 group to T2a and T2b imposes a occupancy restriction of this groups, 12 atoms of Al and 12 atoms of Si in each group. The restriction can be overcome to some extent by setting the number of Al and Si atoms for each group manually with `-p` command. The parameter is discussed below.

Disorder exploration in $FeSbO_4$: Comparison with SOD code.

The $FeSbO_4$ system was explored with the SOD program in ref. [1]. This compound has cation disorder on a single octahedral MO_6 site. The results of the SOD program reported in ref. [1] are reproduced here in table 1. Applying the *supercell* program with the symmetry-merging (`-m`) option for the same supercell sizes gives exactly the same number of symmetry operations, total number of combinations and number of independent configurations as with the SOD code.

The *supercell* command whose output is used to generate a row (for example for supercell size $2 \times 2 \times 1$) in table 1 is of the form:

```
supercell -d -i FeSbO4.cif -s 2x2x1 -m
```

where `-d` (`--dry-run`) options switches the program to dry-run mode (no output-file generation), `-i` (`--input`) must be followed by the input file name (required), `-s` (`--cell-size`) sets the cell size in format `AxBxC`, where `A`, `B` and `C` are positive integer numbers.

Table 1 can be generated automatically (except information about SOD run time) with the script `df_chg.bash` in `supercell/data/examples/FeSbO4` folder. The output of *supercell* is parsed during the execution of the script to extract the relevant information.

As mentioned above this example does not generate output structures. To do so, the user should first create a directory where the structures will be stored and then execute the same type of *supercell* command without the `-d`, and with the `-o <path_to_new_directory>/<ouput_file_prefix>` command instead, as in the example below:

```
mkdir FeSbO4_cell221/
supercell -i FeSbO4.cif -s 2x2x1 -m -o FeSbO4_cell221/FeSbO4_cell221
```

Coulomb energy calculations in γ-Fe_2O_3.

The case of the γ-Fe_2O_3 system, investigated in ref. [2], was also examined. The input structure with name `Fe2O3-P4332.cif` can be found in folder `supercell/data/examples/gamma-Fe2O3/`. Detailed information about the configurations and their degeneracies was presented therein for a supercell $1 \times 1 \times 3$ of γ-Fe_2O_3, all

Table 1: Total number of possible combination of atoms for different supercell sizes of $FeSbO_4$ for comparison with the results published in ref. [1].

Cell, $a\times\ b\ \times\ c$	Number of symmetry operations	Total number of configurations	Number of unique configurations	*supercell*/SOD run time
$1\times1\times1^*$	16	2	1	<1 s
$1\times1\times2^*$	32	6	2	<1 s
$1\times1\times3^*$	48	20	3	<1 s
$1\times1\times4^*$	64	70	8	<1 s
$2\times2\times1^*$	64	70	7	<1 s
$2\times2\times2^*$	128	12870	180	1 s/8.0 s
$2\times2\times3$	192	2704156	15565	7.5 s/7 days
$2\times3\times3$	144	$9\cdot10^9$	N/A	N/A
$3\times3\times3$	432	$1.9\cdot10^{15}$	N/A	N/A

*The data is also presented in table 1 in ref. [1] and fully agrees with *supercell* results.

of which were perfectly reproduced by the *supercell* program.[1]. Among other things, the correlation between total and Coulomb energies was examined in their paper. It was shown that, at least for this particular system, a quite strong correlation is obtained, such that Coulomb energy can be used to approximate the total energy with a reasonable precision. Electrostatic energies can be calculated directly with *supercell*, provided charges are provided for all sites in the CIF input file (with tag `_atom_type_oxidation_number`) and/or in the command line. The following command should be used to calculate the electrostatic energy of all explored unique configurations for supercell size $1\times1\times3$ (examined in ref. [2]):

```
mkdir cell113_out/
supercell -i Fe2O3-P4332.cif -s 1x1x3 -m -q -v 2 -o cell113_out/cell113
```

where the `-q` activates electrostatic energy calculations. This option requires the cell to be charge-balanced, and the calculation of all electrostatic energies will take approximately 5 min. A new file named `cell113_out/cell113_coulomb_energy.txt` is generated, which contains the electrostatic energy of each structure processed. The `_chemical_name_common` parameter value in each output CIF file also contains the energy value.

We can go further and explore the cell $1\times2\times3$ of γ-Fe_2O_3. A fast dry-run of *supercell* program:

```
supercell -d -i Fe2O3-P4332.cif -s 1x2x3 -m -v 2
```

provides information on the total number of combinations 735 471 and the number of unique ones 30 834. The number of configurations is too high to process them all, but they can nevertheless be sampled. The random sampling was discussed above. Here we also extract the structures with lowest (`-n l<number>`) and highest (`-n h<number>`) electrostatic-energies. All possible samplings can be done simultaneously.

```
mkdir cell123_out/
supercell -i Fe2O3-P4332.cif -s 1x2x3 -v 2 -m -q -n l50 -n r100 -n h20 -o
 ↪  cell123_out/cell123
```

Options `-n l50`, `-n h20` and `-n r100` forces program to output only the 50 structures with lowest Coulomb energy, the first 20 structures with highest Coulomb energy and 100 random structures, respectively. The `-q` option is of course mandatory to perform such Coulomb-energy-based samplings.

[1]Table 2 in [2] has a mistyping: L1L3L5L10 degeneracy should be 24 and space group for configuration L1L2L4L5 should be *C2*.

It is important to keep in mind that the output text file `cell123_coulomb_energy.txt` will list the Coulomb energies of all configurations, and not only of the sampled. If you are not interested in all configurations energies, but only in the sampled one, you can find useful files `cell123_coulomb_energy_r.txt`, `cell123_coulomb_energy_l.txt`, and `cell123_coulomb_energy_h.txt`, which lists only the Coulomb energies of configurations sampled with each method. The most of the popular filesystems, has a significant performance degradation, when the total number of file in the folder more than 10 000. To overcome this limitation, the archiving option (`-a`), which will automatically compress the output files, can be used.

```
supercell -i Fe2O3-P4332.cif -s 1x2x3 -m -q -v 2 -o Fe2O3/cell123 -a Fe2O3_cell123.zip
```

This run of *supercell* compress all the output files (except `cell123_coulomb_energy.txt`) to `Fe2O3_cell123.zip` archive. The files will be located in the folder `Fe2O3` within this archive. The name of the files will start from `cell123`. The possible file formats `.zip`, `.tar`, `.tgz`, `.tar.gz`, `.tar.bz2` and `.tar.xz` should be specified like an extension of archive file. The parameter is optional and requires *libarchive* installed, during program compilation.

Supercell **program verification on the α-$Si_xGe_{1\text{-}x}O_2$ system**

The "disorder" part of the CRYSTAL code has previously been applied to a α-$Si_xGe_{1\text{-}x}O_2$ solid solution based on the α-quartz structure[3]. Repeating the same calculations with the *supercell* program again led to the same total number of configurations, number of distinct configurations, and symmetry of individual structures for all x values. The corresponding table may be generated automatically with the script `df_cfg.bash` located in directory `supercell/data/examples/alpha-SiGeO2/`. The *supercell* commands used in this example are of the form:

```
supercell -i alpha-SiGeO2.cif -s 1x1x2 -p "Si1:p=2" -p "Ge1:p=4" -m -o
 ↪  cell112/Si2/SiGeO2_112-Si2
```

here the `-p` option is used to set the number of Si and Ge atoms occupying the disordered mixed site Si1/Ge1. The multiplicity of this site is 3 (trigonal space group $P3_221$, no. 154), which gives for a supercell of size $1\times1\times2$ a total number of 6 positions and imposes that $0 \leq p(\text{Si1}) \leq 6$ and $p(\text{Ge1}) + p(\text{Si1}) = 6$. The structures are generated and stored in directory `cell112/Si2/`, which is created by the script before the command is run. The number "2" in "Si2" stands here for the population p(Si1), and the script actually creates seven such directories named: `cell112/Si<p(Si1)>/` where $p(\text{Si1}) = 0...6$ which contain the generated structures.

Supercell **program verification on the $Cu_2ZnSnS_xSe_{4\text{-}x}$ system.**

Another verification was done by comparison with data published on $Cu_2ZnSnS_xSe_{4\text{-}x}$[4], in which the authors performed the symmetry search manually, rather than with any of the software discussed above. The data can therefore be treated as one more independent evidence of the correctness of our algorithm, which again provides results in complete agreement with those reported in ref. [4]. This example is again distributed with the source code of the *supercell* program, along with a script (`df_cfg.bash` in directory `supercell/data/examples/Cu2ZnSnSxSe4-x/`) that will automatically generate the corresponding tables for direct comparison with the cited article. The *supercell* commands used in this example are of the form:

```
supercell -i stannite.cif -s 1x1x1 -p "S:p=3" -p "Se:p=5" -m -o
 ↪  <output_directory>/stannite
```

in which, as in the previous example, the `<output_directory>` is created before the command is run and the populations of S and Se atoms on crystallographic sites "S" and "Se" are specified with the `-p` option. It is important to keep in mind that "S" and "Se" in "S:p=3" and "Se:p=5" arguments refer to the *crystallographic labels* as they are degined in the CIF file rather than to atom symbols (more typical labels would be of the form "S1" and "Se1"). The multiplicity of these overlapping sites in the cell being 8 and the supercell size being $1 \times 1 \times 1$, the population values "p" should obey $0 \leq p(\text{S}) \leq 8$ and $p(\text{Se}) + p(\text{S}) = 8$.

Supercell **program verification on piezoelectric ceramics $PbZr_xTi_{1\text{-}x}O_3$ (PZT).**

Disorder in piezoelectric ceramics can be also processed with *supercell* program. The well-known $PbZr_xTi_{1\text{-}x}O_3$ (PZT) ceramics are a particularly interesting because of the displacement disorder on the Pb position, which is induced by Zr−Ti substitution and crucially affects its electric and mechanical properties. The example described here is based on Ref. [5], where authors used DFT calculations to investigate the correlation between

substitution ordering in supercell models and the physical properties of the system. They used in this particular case a strongly anisotropic $4 \times 2 \times 1$ supercell of composition $PbZr_{0.5}Ti_{0.5}O_3$. The approximations leads to 10 symmetry unique configurations, which were all presented in the paper. They can be easily be generated with the *supercell* program embedded in a simple script `df_cfg.bash` located in `supercell/data/examples/PZT/` folder. We note that the order of configurations differs in the script and in the paper. The command line used in this script is:

```
supercell  -s 4x2x1 -i PZT-PbZr05Ti05O3.cif -m -o <output_directory>/PZT421
```

Using the *supercell* program makes it possible to proceed with larger supercell sizes $4 \times 2 \times 2$ and $4 \times 3 \times 2$, yielding 490 and 29606 unique configurations, respectively. The second case may be difficult to process, but the first one is absolutely feasible. Such supercell size allow to research structure distortion in z-dimension also.

Disorder in $Pb_{0.5}Sn_{0.5}Te$: calculation of atom-pair correlation functions

The purpose of this section is to describe the procedure and results of the exploration of the $Pb_{0.5}Sn_{0.5}Te$ system discussed in the main text, in which we use the *supercell* program combined with a structure-analysis tool (the GULP program) to calculate atom-pair correlation functions. These functions are then used to evaluate to what extent the generated structures are representative of random Pb/Se disorder through their adequacy with Special-Quasirandom-Structure (SQS) character[6].

This example is performed automatically with the script `df_cfg.bash` located in directory `supercell/data/examples/PbSnTe-SQS/` for two supercell sizes: $1 \times 1 \times 2$ and $1 \times 2 \times 2$. The script uses *supercell* commands such as:

```
supercell -i PbSnTe2.cif -s 1x1x2 -m -o cell_1x1x2/PbSnTe_1x1x2
```

The GULP program is then used to perform the following structure analysis on each one of the (symmetry-unique since the `-m` option is used) output configurations. The correlation functions are calculated like a total energy of the system with set of "fake" potentials. The potential energy it set to +1 for A—A and B—B interactions and -1 for A—B at distance R_m, with m being the coordination sphere number (see main text), and zero for all other distances. The result is saved in text files `SQS-1x1x2` and `SQS-1x1x2` in the form of tables which list, for each configuration:

- the structure name (ex. `cell_1x1x2/PbSnTe_c1x1x2_i<index>_w<weight>.cif`)
- the atom-pair correlation coefficients (see main text) calculated for the first 4 shells of each cationic site (ignoring Te atoms), which correspond to A—B distances (where A, B = Pb or Sn) of 4.51, 6.39, 7.83, and 9.04 Å, as listed in the file header.

The adequacy of the structure to the SQS criteria is indicated by how close these four correlation functions, and in particular the first three, are to zero (because the substituted Pb and Sn atoms are present in equal concentrations, see main text). A good way to visualize these results is for example to sort the structures by ascending order of the absolute value of those coefficients in columns 1, 2, 3, and 4 (in this order).

Permutations in ice I_h $1 \times 1 \times 2$ supercell.

The method described in the main paper to generate correlated ice I_h structures will not work with supercells larger than the original one. An advanced method to do so is therefore presented here. The input CIF file should be changed manually to use this method. We strongly encourage the user to also check the `df_cfg.bash` script in folder `supercell/data/examples/ice-Ih-adv/`, which implements this procedure.

As mentioned in the main text, the ordering of H atoms in this system is governed by two restrictions: the coordination number of all O atoms should be two and H atoms should not be in close contact with each other. Further below, we describe how to impose the first restriction in the initial structure, by grouping atoms in a non-standard way. But first of all, we generate a $1 \times 1 \times 2$ supercell, keeping the disorder:

```
supercell -i ice-Ih.cif -s 1x1x2 -p "*:fixed" -o ice-Ih-121-adv.cif
```

where the partial occupancies on both sites are kept fixed. (This step could of course alternatively be done with any program for the visualization of crystallographic structures.) The output structure is shown on fig. 1. Each oxygen atom is still surrounded by 4 hydrogen positions, strictly two of which should be occupied by H atoms in the end. In the figure, the hydrogen sites are associated with different O atoms marked with different

Figure 1: Crystal structure of ice I_h. Cell 1x1x2. Hydrogen sites connected to the same oxygen atoms are displayed with the same color, each color being associated in this initial structure to a different H-atom label (from H1 to H8).

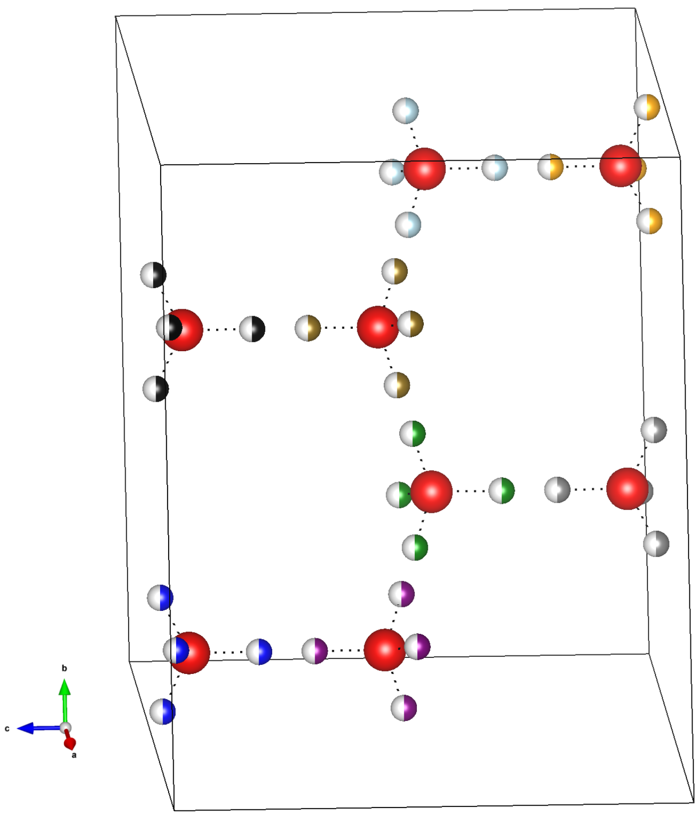

colors. The H atom labels are then changed manually in the same way as the color code, ensuring that (i) all hydrogen positions attached to the same O atom have the same label and that (ii) hydrogen atoms positions associated with different O atoms have different labels (ex. H1, H2, H3, etc).

Importantly, the *supercell* program will associate sites with identical crystallographic labels within a common group (as defined in the main text), such that a run with this customized file (`supercell/data/examples/ice-Ih-adv/ice-Ih-121-adv.cif`) as an input gives 8 groups with $C_4^2 = 6$ combinations each, which yields a total of 6^8 =1 679 616 configurations. The *supercell* command used for this step is the following:

```
supercell -i ice-Ih-121-adv.cif -m -q -p "O:c=-2" -p "H*:c=+1" -p "r(H[5-8]):fixed" -o
↪  <output_directory>/ice-Ih-l1
```

where the `-p --property` option is first used to set the charges (`<label>:c=<charge_value>`) of O and H crystallographic sites for the Coulomb energy calculation (`-q` option). The "`H*`" notation here sets the specified charge to all sites starting with "H". The `-p` option is then used again to fix the partial occupancies of H sites labelled "H5" to "H8" with the notation "`r(H[5-8]):fixed`", which excludes these sites from the permutation. Here the "r" means that the expression inside the parentheses should be interpreted like a Perl regular expression (RegEx). A description of the corresponding syntax can be found at `http://en.wikipedia.org/wiki/Regular_expression`. More information on these wild cards and other advanced text search functionalities to set the properties of several crystallographic sites are provided in the manual (`man supercell`). By comparison, applying the *supercell* program to the same supercell size with the original grouping gives a total of 189 290 920 configurations, which is approximately one hundred times more than with the customized CIF file.

This new group assignment not only solves the problem of the O coordination, but also splits the 32 H positions into 8 groups of sites. The number of configurations (1.6 million) is still very high to generate them all simultaneously, but the process can be done step-by-step. We note that such a number of configurations is not a problem for the*supercell* algorithm, which can deal with up to 8×10^8 configurations, but for storage and data analysis (by the GULP program). 4 out of 8 groups can be fixed in a first step to generate 656 (after

symmetry merging) output structures. These files can then be analyzed to exclude those with H−H close contact. Only valid structures are kept for the next step, where they are used as new inputs for the *supercell* program, with the command:

```
supercell -i <new_input_structure> -m -q -p "O:c=-2" -p "H*:c=+1"  -o
 ↪  <output_directory>/<prefix>
```

The process gives only 9 structures which obey both conditions, and which may be found in the ice-Ih-cfgs-final/ directory.

The directories and files created for this example by the script: df_cfg.bash in folder supercell/data/examples/ice-Ih-adv/ are organized as follows:

- ice-Ih-121-adv.cif: the starting structure, based on a $1\times2\times1$ supercell of the original ice I_h structure, with labels modified as described above.
- ice-Ih.gin_template: contains the input-file template for the structure analysis performed with Gulp, which lists inter-atomic distances and is parsed to locate H-H close contacts and check the O-atom coordination.
- ice-Ih-cfgs-l1/: contains partially-disordered configurations files with names ice-Ih-l1_i<index>_w<weight>.cif, in which permutations were treated on groups of H sites H1 to H4, but where partial occupancies were kept on groups H5 to H8. Each structure is associated with a folder (ice-Ih-cfgs-l1/ice-Ih-l1_i<index>_w<weight>/gulp/) which contains the output of the structure analysis performed with the GULP program.
- ice-Ih-cfgs-l2/: Contains copies the structures satisfying both restrictions on the local structure (no H−H close contact and 2-coordinated O) among those obtained during the first step. Each of these structures are then associated with directories of the type:
- ice-Ih-cfgs-l2/ice-Ih-l1_i<index>_w<weight>/ which contains the structures generated by the second run of the algorithm, where the remaining disordered sites (H5 to H8) are treated. These structures are named ice-Ih-l1_i<step1_index>_w<step1_weight>-l2_i<step2_index>w_<step2_weight>.cif and are again associated with all folder (same name without the extension) containing the result of the structure analysis.
- ice-Ih-cfgs-final/: contains the final selection of structures with disorder in all H sites treated, and which satisfy both structure-restriction conditions.

Supercell program integration to research process.

Our program is "link in chain" and the efficiency of the research process, obviously, depend on another links and connection between them. Below, there is a list of the programs/resources (in addition to discussed in the main paper), which can be possibly used with supercell program in disorder compound research.

ASE (https://wiki.fysik.dtu.dk/ase/). Atomic Simulation Environment is another set of python tool for setting up, manipulating, running, visualizing and analyzing atomistic simulations. Support many calculation software, both classical and *ab-initio*, including CASTEP and VASP.

cif2cell (http://sourceforge.net/projects/cif2cell/). Cif2cell is a tool to generate an input structure in different formats, like CASTEP, CP2K, CRYSTAL09, Quantum Espresso, VASP and many more.

COD (http://www.crystallography.net). Open-access structural database with more than 356 000 records (Feb 2016).

GULP (http://gulp.curtin.edu.au/). GULP is force-field program for performing a variety of tasks on a range of system types. It can be useful for optimizations, energy calculation and analysis of the structure.

EnCIFer (http://www.ccdc.cam.ac.uk/Community/freeservices/encifer/). A GUI tool for validating of *supercell* input structures. Useful for draft structures.

VESTA (http://jp-minerals.org/vesta/). A powerful tool for visualization and editing of crystallographic structures. It supports many input/output format (including cif) and can visualize partially occupied sites.

References

[1] R Grau-Crespo, S Hamad, C R a Catlow, and N H De Leeuw. Symmetry-adapted configurational modelling of fractional site occupancy in solids. *Journal of Physics: Condensed Matter*, 19(25):256201, June 2007.

[2] Ricardo Grau-Crespo, Asmaa Y Al-Baitai, Iman Saadoune, and Nora H De Leeuw. Vacancy ordering and electronic structure of γ-Fe_2O_3 (maghemite): a theoretical investigation. *Journal of physics. Condensed matter : an Institute of Physics journal*, 22(25):255401, June 2010.

[3] Kh E El-Kelany, a Erba, P Carbonnière, and M Rérat. Piezoelectric, elastic, structural and dielectric properties of the $Si_{1\text{-}x}Ge_xO_2$ solid solution: a theoretical study. *Journal of physics. Condensed matter : an Institute of Physics journal*, 26(20):205401, 2014.

[4] Chaochao Dun, N. a W Holzwarth, Yuan Li, Wenxiao Huang, and David L. Carroll. $Cu_2ZnSnS_xO_{4\text{-}x}$ and $Cu_2ZnSnS_xSe_{4\text{-}x}$: First principles simulations of optimal alloy configurations and their energies. *Journal of Applied Physics*, 115(19), 2014.

[5] Ilya Grinberg, Valentino R. Cooper, and Andrew M. Rappe. Oxide chemistry and local structure of $PbZr_xTi_{1\text{-}x}O_3$ studied by density-functional theory supercell calculations. *Physical Review B - Condensed Matter and Materials Physics*, 69(14):1–17, 2004.

[6] Alex Zunger, S. H. Wei, L. G. Ferreira, and James E. Bernard. Special quasirandom structures. *Physical Review Letters*, 65(3):353–356, 1990.
