# Supplementary material for: Supercell program: a combinatorial structure-generation approach for the local-level modeling of atomic substitutions and partial occupancies in crystals
Source: J Cheminform. 2016 Mar 31;8:17. doi: 10.1186/s13321-016-0129-3 (PMC4818540; doi:10.1186/s13321-016-0129-3)
Supplement: Supplementary file 2 — 10.1186/s13321-016-0129-3 supercell.zip --- The snapshot of supercell code from github. [file 13321_2016_129_MOESM2_ESM.tar › supercell/doc/supercell_man.pdf]

supercell
—
A Scientific Tool

Kirill Okhotnikov, Sylvian Cadars

September 1, 2015
Version 1.0

Abstract

supercell is a tool to convert a crystallographic structure with partial occupancy and/or vacancies to ordinary supercell structure suitable for calculations.

1 Synopsis

supercell **-h, --help**

supercell [**OPTIONS**] **-i** *input-file*

supercell [**OPTIONS**] **--input=***input-file*

2 Description

Disordered compounds are very important now for fundamental science and industrial applications, but most of the methods of exploring solid state material properties required ideal periodicity. In opposite of many reported research, which implies periodicity to disordered system "by hand" and ad-hoc the program *supercell* allows to apply the approximation systematically with all-in-one algorithms implemented for structure manipulation, supercell generation, permutations of atoms and vacancies, charge-balancing, detecting symmetry equivalent structures, Coulomb energy calculations and sampling output data. The advantages of the program is fast-algorithms implementation and availability under GPL license.

3 Options

-h, --help Print help message and exit the program.

-v *level*, --verbose=*level* Change verbosity level from default 1. Level 0 - quiet mode, only error output. Level 1 suggested for regular users. Higher levels suggested to developers, bug tracking and long program execution times.

-i *input-file*, --input=*input-file* Required option. Input file in CIF format. Only one file can be specified.

-d, --dry-run The option is highly recommended for the first run of the program with new input specified. With the option the program will do everything but write the files. Be careful if you switched **-m**, **--merge-by-distance** option: the program will go through all crystal structures, so even the dry-run mode can take time.

-s *cell-size*, --cell-size=*cell-size* The option specifies the size of the supercell. The format is AxBxC, where A, B, C are positive integer multipliers of **a**, **b** and **c** unit cell vectors of input system. Default is ”1x1x1”.

-c *balance-type*, --charge-balance=*balance-type* The option helps you to charge balance the system. Be careful, charge balancing with wrong input charges will make output system composition far from desired or it even can freeze the program. Charges (i.e. oxydation states) may be specified with the **-p**, **--labels-properties** option (see below) or directly in the CIF file (”_atom_type_oxidation_number”). The possible arguments are:

- no No charge balancing. The option will set all charges to zero.
- try Default. Try to charge balance system, if initial system is not charged.
- yes Charge balance the system.

-p *labels-properties*, --property=*labels-properties* The option will allow you to manually specify some properties of atoms with specific labels. You may use the simple or the extended syntax.

- Simple: <label>:<property_name>[=<property_value>].
- Extended: ”<OPT>(<labels>):<properties>” (Do not forget to put extended syntax in quotes).

Where <label> is the label of the crystallographic site, the properties of which you want to change, <labels> set of space-separated labels. <OPT> is the type of string-pprocessing you want use to set the labels. The possible values of <OPT> are:

- p Treat the labels in parenthesis like a plain string.
- w, ”” Default for simple syntax. Treat the labels in parenthesis like a wild card. For example, N* means all labels, starting with N: N1, N12, but also Na3. You can use * (any numbers of any symbol) and ? (any symbol: exactly one).
- r Treat the labels in parenthesis like a Perl Regex. Syntax description can be found at `http://en.wikipedia.org/wiki/Regular_expression`.

The <property_name> is the name of the property you want to set for all specified crystallographic sites. Some properties can have values, which you should set using equal symbol. The properties can be:

- c[harge] Set the charge of atoms with specified label(s). Floating-point value in elementary charge units.
- p[opulation] Number of atoms with specific label(s) in output supercell structure.
- [not]fixed Exclude crystallographic sites from the combinatorial analysis. The output supercell file will contain partial occupancies for the corresponding sites (if there were partial occupancies in the original structures).

Some fancy examples can be found below.

-t *tolerance*, --tolerance=*tolerance* The argument of the option specifies the maximum distance (in Angstroms) between sites that should be contained within the same group (meaning that the corresponding positions in the supercell cannot be occupied simultaneously). Check carefully the minimal distance between 2 atoms assigned to different groups, which is written in the output of *supercell* before changing this parameter. Default *tolerance*=0.75 A.

-m, --merge-by-distance The options enables the symmetry-check algorithm on output structures. Structures that can be transform to each other using crystallographic symmetry operations will be merged and stored as one structure. For cases with more than 10^4 total combinations it is recommended to use verbosity level 2 or higher to trace program execution.

-n *selection*, --store-structures=*selection* The option allows you to generate subsets of structures (rather than all) according to certain criteria (listed below). The option is very useful to both save disk space and facilitate navigation among files for large numbers combinations (typically more than 10^5).The argument of the option has special structure <sampling type><count>. The <count> is a positive integer value or zero. <sampling type> is a letter which sets the sampling (selection) mode:

r : select <count> random structures.

f : select <count> first structures.

a : select <count> last structures.

l : select <count> low energy structures (**--coulomb-energy** option required).

h : select <count> high energy structures (**--coulomb-energy** option required).

Example: l100 will store first 100 structures with low Coulomb energy.

Multiple declaration of the option is allowed. If the option is enabled, an extra prefix (the letter <sampling type> will be added to output file name. Be careful, enabling the option required an extra memory, proportionally to the number of structures to store.

-q, --coulomb-energy The option enable Coulumb energy calculations. The result energies for each structures are stored in file <output-prefix>_coulomb_energy.txt. The energies can help to consider the relevant regular structures for following calculations. Ewald summation algorithm is used for the calculations. Be careful, in case of charged cell the energy values can be meanless. See also **--charge-balance** option.

-o *output-prefix*, --output-prefix=*output-prefix* The options specify output file name prefix. The prefix can contain folder name but the folder should be created before run the program. For example, --output-prefix=myfolder/myfiles. The output files will be created according to templates. For non merging run the template will be <output-prefix>_i<sampling type><index>.cif. For merging run the template will be <output-prefix>_i<sampling type><index>_w<weight>.cif, where <weight> - number of the structures merged to the structure. Be careful, all existing files with mask "<output-prefix>*.cif" will be deleted during not dry-run. The <output-prefix>_coulomb_energy.txt file will be overwritten only in case of **--coulomb-energy** option enabled.

-a *archive-file*, --archive=*archive-file* The option specify a target archive file for the output files, except energy file for all permuted configurations. If archive-file string is empty (default) no packing will be performed and all files will be stored directly to disk. If the file with the name exist it will be overwriting. This optional feature required *libarchive* `http://www.libarchive.org/` to be available in the system. Otherwise the option will be disabled. Check the status by **-h** option. The extension of the file should be set according to desired archive type: ".zip", ".tar", ".tgz", ".tar.gz", ".tar.bz2", ".tar.xz".

4 Files

5 Examples

The examples are based on file `supercell/data/examples/Ca2Al2SiO7`.

- Dry run. Obtain information about the structure: group assignment, cell size, etc. It is good practice to start a new structure processing with this command. This run doesn't produce any output files.

  supercell -d -i Ca2Al2SiO7.cif

- Dry run. Obtain information about the structure.

  supercell -d -i Ca2Al2SiO7.cif -s 1x1x2

- Dry run. Obtain information about the structure.
  supercell -d -i Ca2Al2SiO7.cif -s 1x1x2

Some advances examples, with *supercell* embedded to bash scripts you can find in `supercell/data/examples` folder.

6 Requirements

openbabel Required.

libeigen Required.

libarchive Optional.

7 Bugs and limitations

Supercell size limitation. The maximum number of permutations should not exceed limit of 800 million. If more, the program will return a error. If the number of permutations within the groups is less than the limit, but total number of combinations is above the limit, the pro gram can be executed step-by-step with some groups excluded from permutation. Due to rapid increasing of number of combination with changing cell size the program can crash with arithmetic overflow at large cell, when number of combination more than 2^{63} (around 10^{18}) due to finite size integer variables in the program.

Filesystem limitation. Although, *supercell* can produce millions of files, most of the filesystems can't manage more than one thousand files in one folder with acceptable performance. Be careful, if you need to process a lot of combination, sample them or use **-a** option.

Symmetry information handling in input file. Our program used *openbabel* libraries to handle symmetry operations in input file. The algorithm, implemented there, gives wrong result for some structures. The problem can be fully solved by converting input file to P1 structure with any other software. In most of the cases, the problem can be solved by removing "_space_group_IT_number" tag from input cif file.

8 See Also

openbabel(1).

9 Version

Version: 1.0 of September 1, 2015.

10 License and Copyright

Copyright All rights to the program belongs to authors.

License This program can be redistributed and/or modified under the terms of the GNU GENERAL PUBLIC LICENSE Version 2.

Misc The actual version of *supercell* may be found on my homepage
`https://github.com/orex/supercell`.

11 Author

Kirill Okhotnikov e-mail: `kirill.okhotnikov@gmail.com`

Dr. Sylvian Cadars e-mail: `sylvian.cadars@cnrs-imn.fr`
Institut des Materiaux Jean Rouxel (IMN) - UMR6502
2 rue de la Houssiniere, BP32229
44322 Nantes cdx3, France
Tel: +33 (0)2 40 37 39 34
Fax: +33 (0)2 40 37 39 95
